# Supplementary material for: Neurotransmitter Funneling Optimizes Glutamate Receptor Kinetics
Source: Neuron. 2018 Jan 3;97(1):139–149.e4. doi: 10.1016/j.neuron.2017.11.024 (PMC5766834; doi:10.1016/j.neuron.2017.11.024)
Supplement: Document S1. Figures S1–S8 and Tables S1–S4 [file mmc1.pdf]

Neuron, Volume *97*

Supplemental Information

Neurotransmitter Funneling Optimizes

Glutamate Receptor Kinetics

Alvin Yu, Héctor Salazar, Andrew J.R. Plested, and Albert Y. Lau

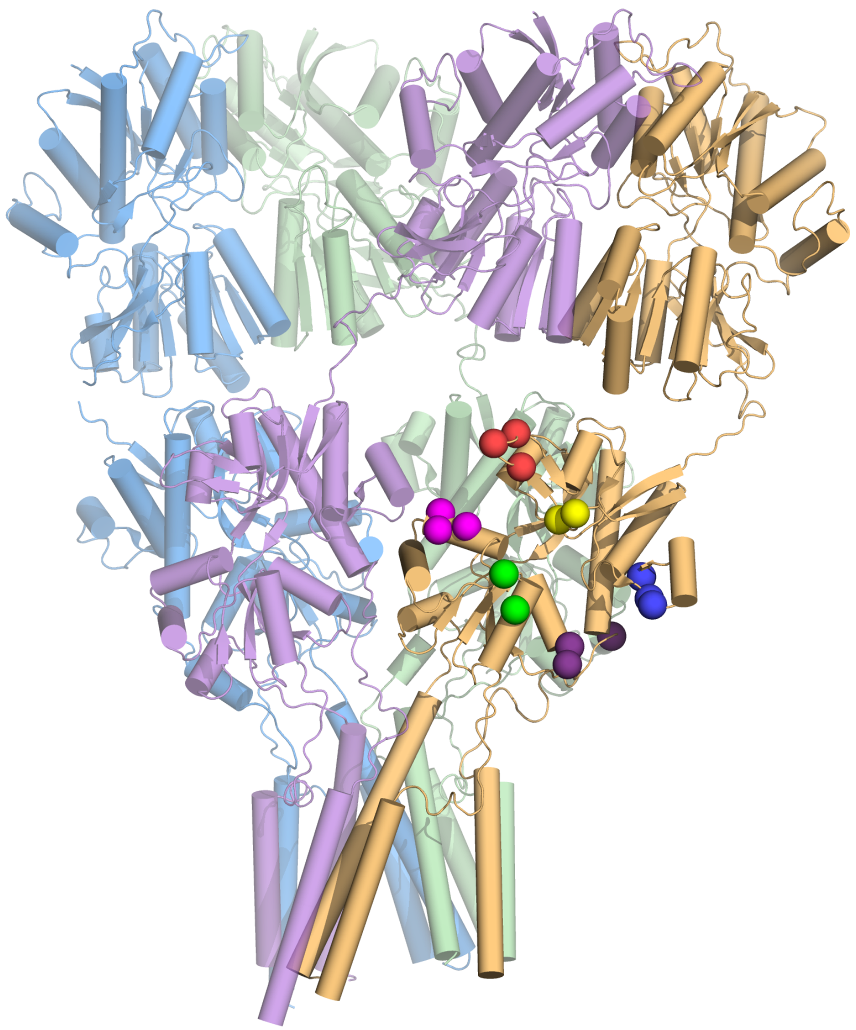

Figure S1. Sites of mutation in a tetrameric GluA2 receptor. Related to Figure 4.

Each sphere corresponds to a site of mutation shown in Figure 4a in a tetrameric receptor. R453, D456, and K458 are in red; E657, R660, and R661 are in magenta; D447 and K449 are in yellow; R684 and E688 are in green; K409, K410, and E422 are in blue; R715, K716, and D769 are in violet. The mutations occur in all subunits of the receptor, but they are shown in only one subunit for clarity. The structure shown is PDB ID 4U2P (Dürr et al., 2014).

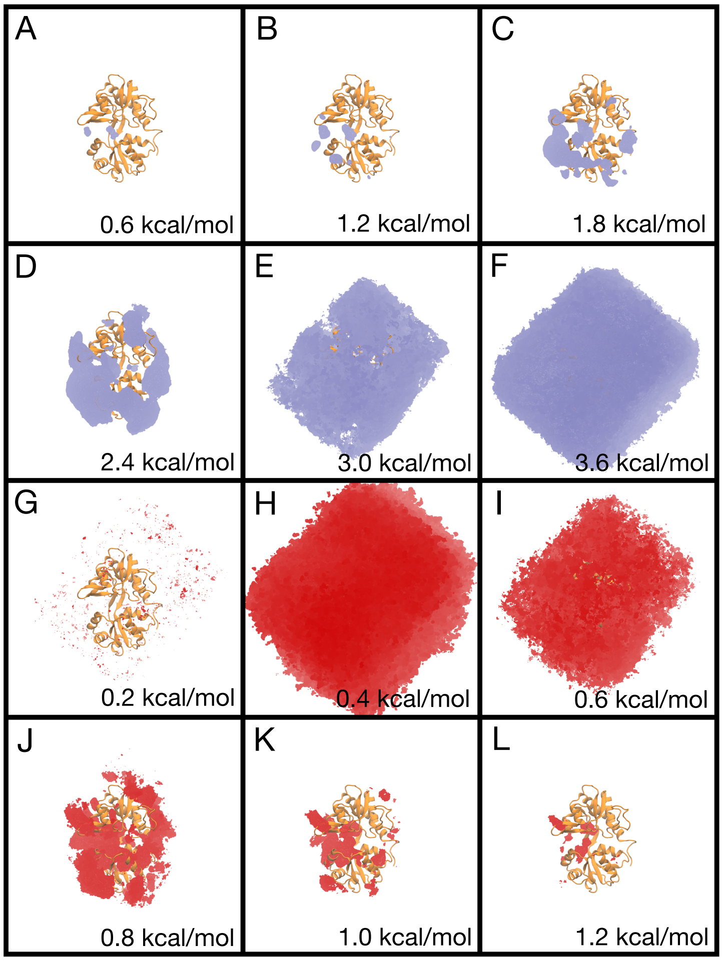

Figure S2. Error analysis of the ligand density PMF. Related to Figure 2. (a–f) The 3D PMF (blue) is shown at contour levels ranging from 0.6 kcal/mol to 3.6 kcal/mol in increments of 0.6 kcal/mol. **(g–l)** The statistical uncertainty in the 3D PMF determined using the approach of block averaging. 10 blocks were used. Contours of the 3D standard deviation (red) are shown from 0.2 kcal/mol to 1.2 kcal/mol in increments of 0.2 kcal/mol. Protein-ligand interactions (a–d) have uncertainties ranging from approximately ±0.8 to ±1.2 kcal/mol (g–l), whereas sites in bulk solvent (e, f) have lower uncertainties of approximately ±0.2 to ±0.6 kcal/mol (j-l).

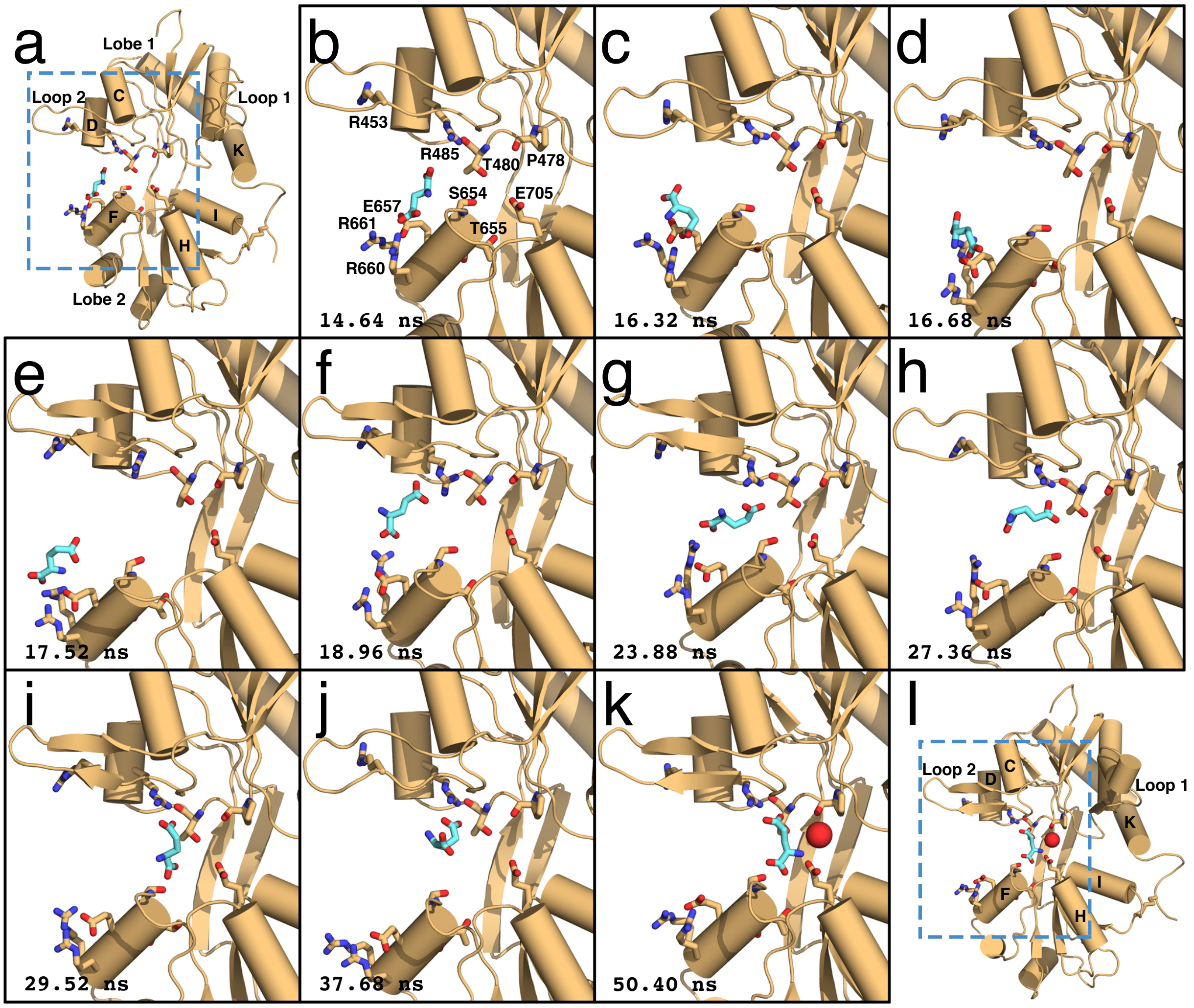

Figure S3. Glutamate binding in the inverted conformation. Related to Figure 3 and Movie S2. Time points (lower left of each panel) are relative to the start of Movie S2. **(a)** Prior to ligand binding, the LBD is closed; (ξ_1, ξ_2) = (11.0, 11.7 Å). The ligand's γ-carboxylate contacts R660. **(b)** Close-up view of **a**. **(c–d)** The ligand moves away from R485 as it is passed from R660 to R661. Interactions between the ligand and helix F residues switch from the γ-carboxylate at R660 to the α-carboxylate at R661. **(e)** The LBD opens, (ξ_1, ξ_2) = (15.0, 11.2 Å), to allow ligand entry into the binding pocket. **(f–g)**

The ligand metastably bridges Lobes 1 and 2 as its γ-carboxylate contacts R485, which has transiently rotated out of the binding pocket to form the interaction. **(h)** Interactions with helix F residues are broken, and the ligand moves into the binding pocket, tethered to R485. **(i)** Glutamate adopts the inverted bound conformation. **(j–k)** The ligand's amide contacts E705. A hydrogen bonding network is formed involving P478, a water molecule, and the ligand's amide, stabilizing the inverted conformation. **(l)** Expanded view of **k**. The LBD remains slightly open around the inverted conformation; (ξ_1, ξ_2) = (13.2, 11.1 Å). This trajectory corresponds to T_{mon1} in Table S1.

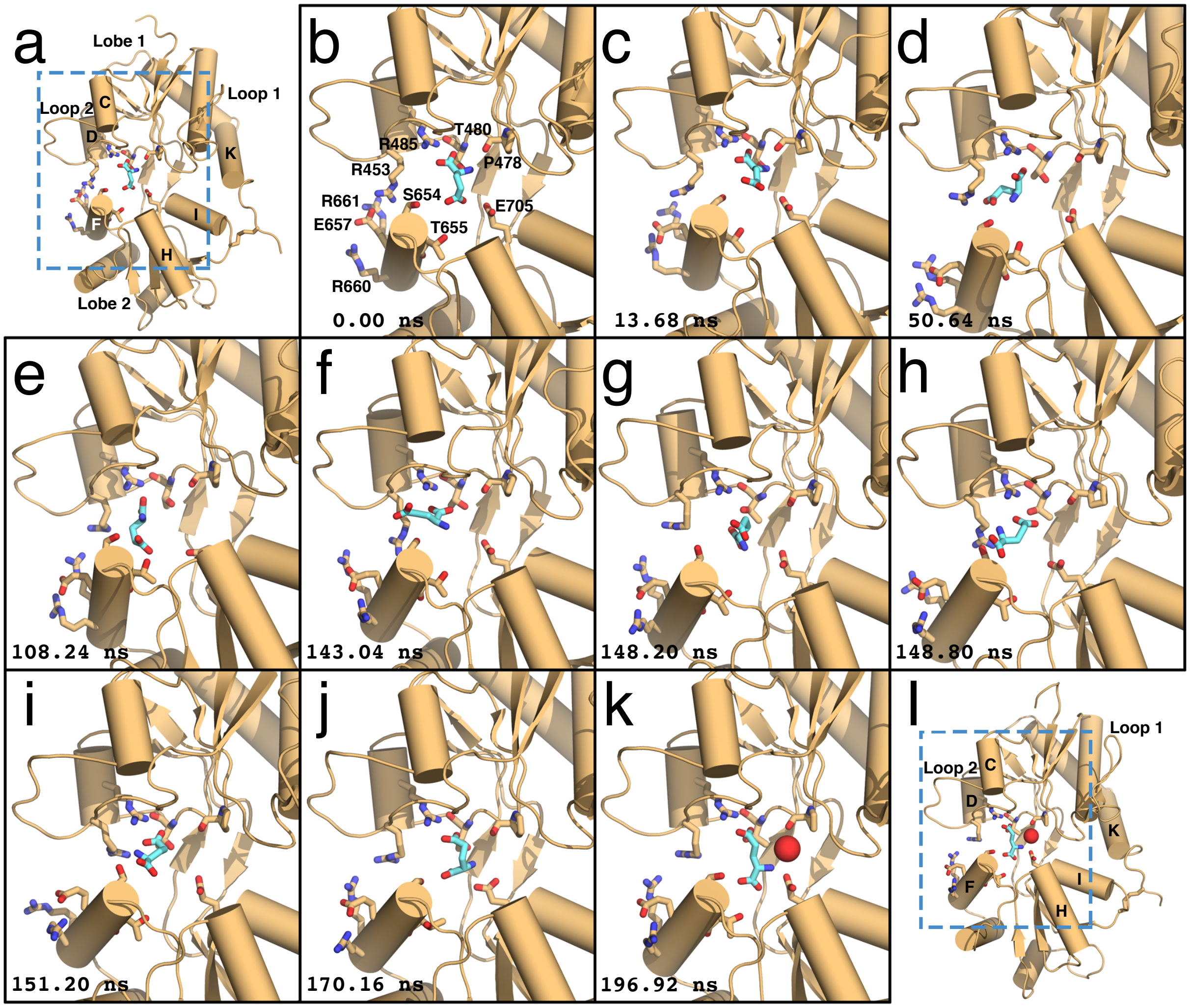

Figure S4. Interconversion between bound ligand conformations. Related to Figure 3 and Movie S3. Time points (lower left of each panel) are relative to the start of Movie S3. **(a)** Initially, the ligand binds in the crystallographic conformation. **(b)** Close-up view of **a**; the ligand's α-carboxylate contacts R485, whereas the amide is coordinated by P478 and E705. **(c–d)** The γ-carboxylate swings out of the binding pocket to contact R453. Interactions between the ligand's amide with P478 and E705 are broken. **(e–f)** The γ-carboxylate rotates freely in the binding cleft while the α-carboxylate remains

tethered to R485. **(g)** Contacts between the α-carboxylate and R485 are severed as the ligand repositions. **(h)** The γ-carboxylate of the ligand contacts R485. **(i)** The ligand swings into the binding pocket. **(j)** Glutamate adopts the inverted bound conformation. **(k)** The ligand's amide contacts E705, and P478 forms a hydrogen bond with a water molecule that stabilizes the inverted conformation. The ligand's α-carboxylate interacts with S654 in Lobe 2. **(l)** Expanded view of **k**. This trajectory corresponds to T_{dim2} in Table S1.

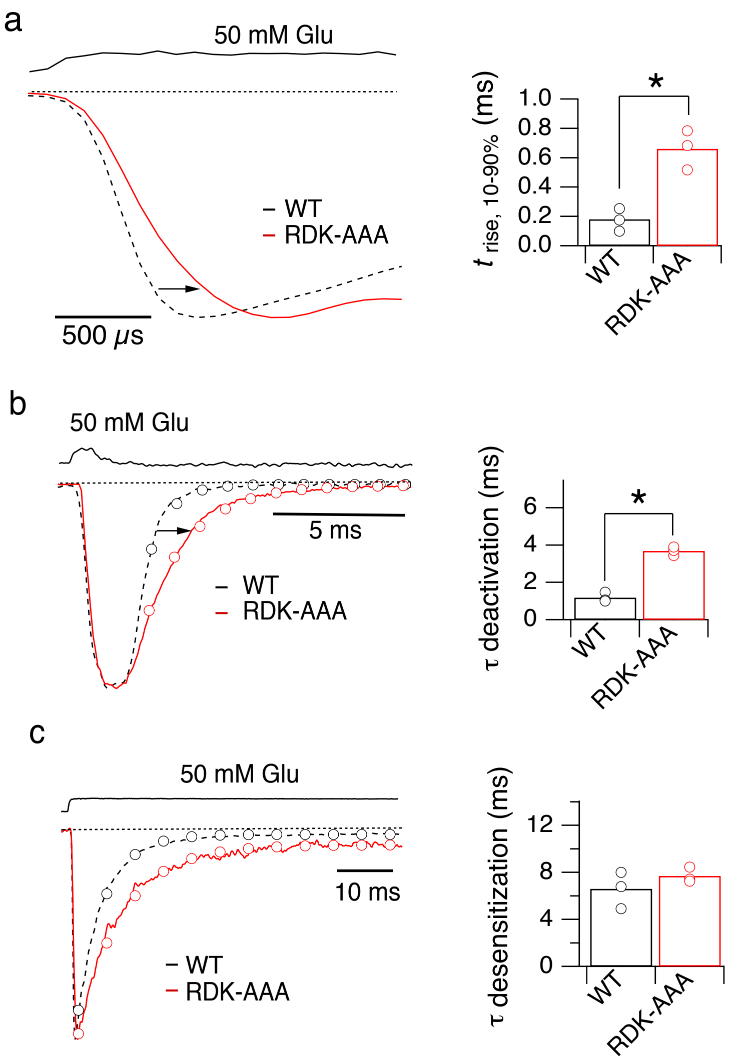

Figure S5. Slower activation and deactivation are retained in high glutamate. Related to Figure 4. (a) Normalized current responses corresponding to activation by a long pulse of 50 mM glutamate are shown for the RDK-AAA mutant (red) with WT GluA2 (dashed black trace). The upper trace shows the application of glutamate. Rise times (10-90%) from individual patches are shown in the bar graph (right panel). (p = 0.01 vs. WT; t-test, n = 3). **(b)** Monoexponential decays for WT GluA2 (dashed black line) and RDK-AAA (red line) were fitted for the deactivation in response to a 1 ms pulse of 50 mM glutamate (open circles). The upper trace shows the open tip response.

Deactivation time constants from individual patches are plotted in the bar graph (right panel; $p = 0.0003$; *t*-test, $n = 3$). **(c)** Desensitization time constants were not altered by the RDK-AAA mutant. Monoexponential fits as in **b**. Desensitization time constants from individual patches plotted in the bar graph (right panel) revealed no difference between WT and RDK-AAA ($p = 0.3$; *t*-test, $n = 3$).

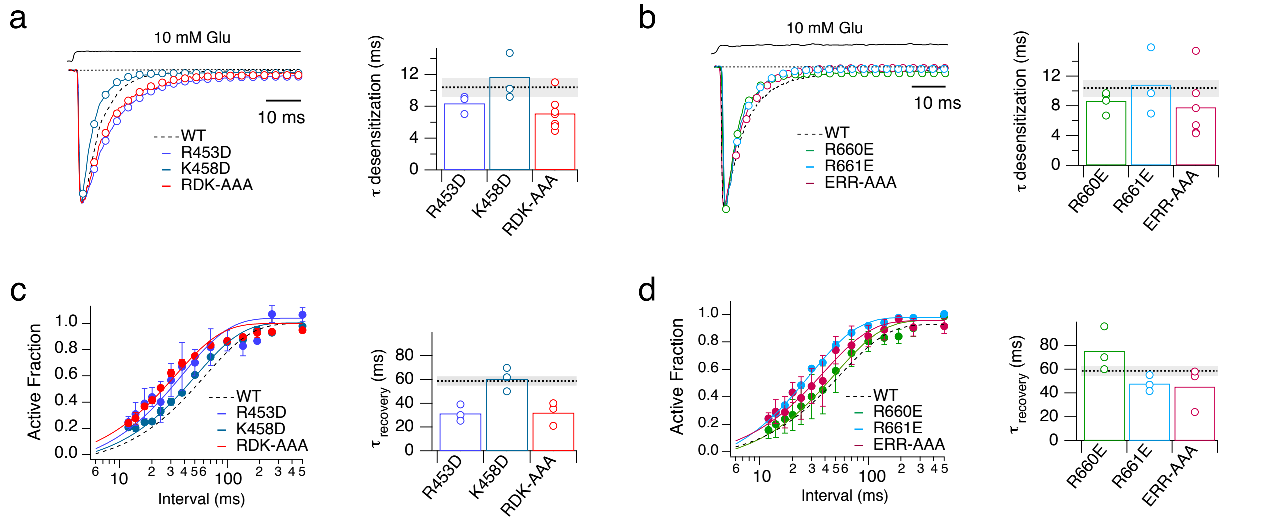

Figure S6. Desensitization and recovery from desensitization of receptors mutated at metastable sites 1 and 2. Related to Figures 4 and 5. (a) Monoexponential fits to desensitization decays of R453D, K458D and the R453D, D456A, K458A (RDK-AAA) mutants in response to 10 mM glutamate were similar to WT ($p > 0.03$; *t*-test, $n = 3 – 7$). A typical WT GluA2 response is shown as a dashed line. Decay constants for individual patches are plotted in the right panel, with the average value for WT GluA2 indicated by a black dashed line, with standard error shaded in light grey. **(b)** As for **a**, but for the R660E, R661E and E657A, R660A, R661A triple mutants. Fits were similar to that of WT ($p > 0.2$; *t*-test, $n = 3$). **(c)** Left panels show fits to pooled responses (“Active Fraction”) at increasing intervals after a long pulse of 10 mM glutamate for WT and the different mutants. The R453D and RDK-AAA mutants recovered from desensitization about twice as fast as WT ($p < 0.02$; *t*-test, $n = 3$). Recovery time constants for individual patches, with the WT mean value and standard error indicated as in **a–b**. **(d)** Recovery data for the R660E, R661E and ERR-AAA

mutants presented as in **c**. Recovery time constants for these mutants were not significantly different from that of WT ($p > 0.1$; *t*-test, $n = 3$).

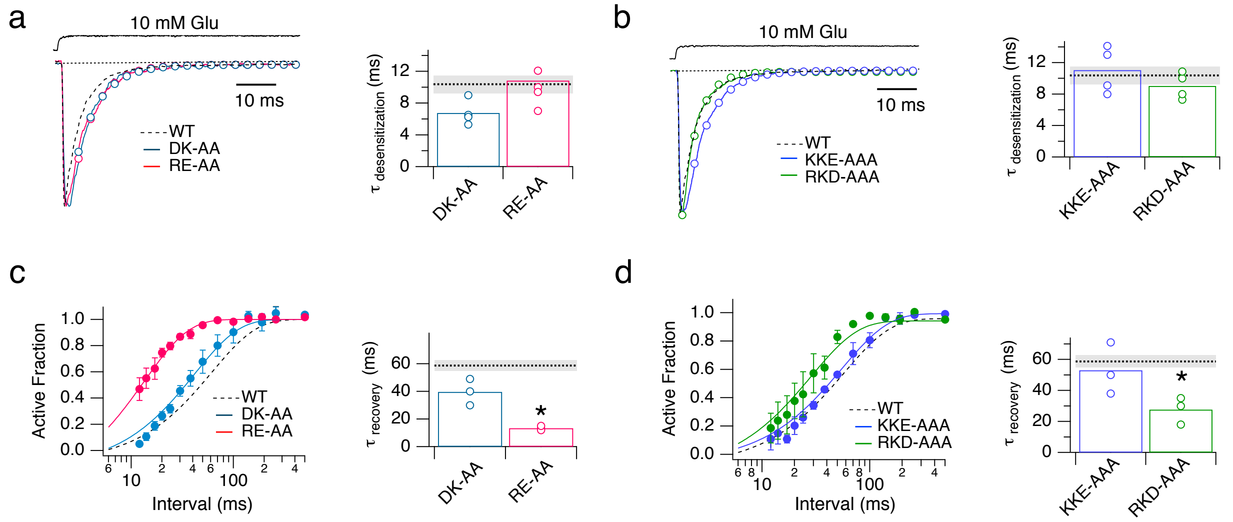

Figure S7. Desensitization and recovery from desensitization of receptors with mutations in Pathway 3 and in off-pathway regions. Related to Figure 6. (a) Monoexponential fits to desensitization decays of DK-AA (D447A K449A) and RE-AA (R684A, E688A) mutants in response to 10 mM glutamate were similar to WT ($p = 0.03$ for DK-AA, $p = 0.8$ for RE-AA; t-test, $n = 3$–7). Fits are represented by open circles. A typical WT GluA2 response is overlaid (black dashed line). Decay constants for individual patches are plotted in the right panel, with the average value for WT GluA2 indicated by a black dotted line, with its standard error shaded in light grey. **(b)** As for **a**, but for the KKE-AAA and RKD-AAA control mutants ($P = 0.7$ and 0.4 vs. WT, respectively; t-test). **(c)** Left panels show fits to pooled responses (“Active Fraction”) at increasing intervals after a long pulse of 10 mM glutamate for WT and the DK-AA and RE-AA mutants. The RE-AA mutant gave very small currents recovered from desensitization about four times as fast as WT ($p = 0.008$; t-test, $n = 3$–5). Recovery time constants for individual patches, with the WT mean value and standard error

indicated as in **a–b**. **(d)** Recovery data for the KKE-AAA and RKD-AAA mutants (P = 0.6 and 0.01 vs. WT, respectively; t-test), presented as in **c**.

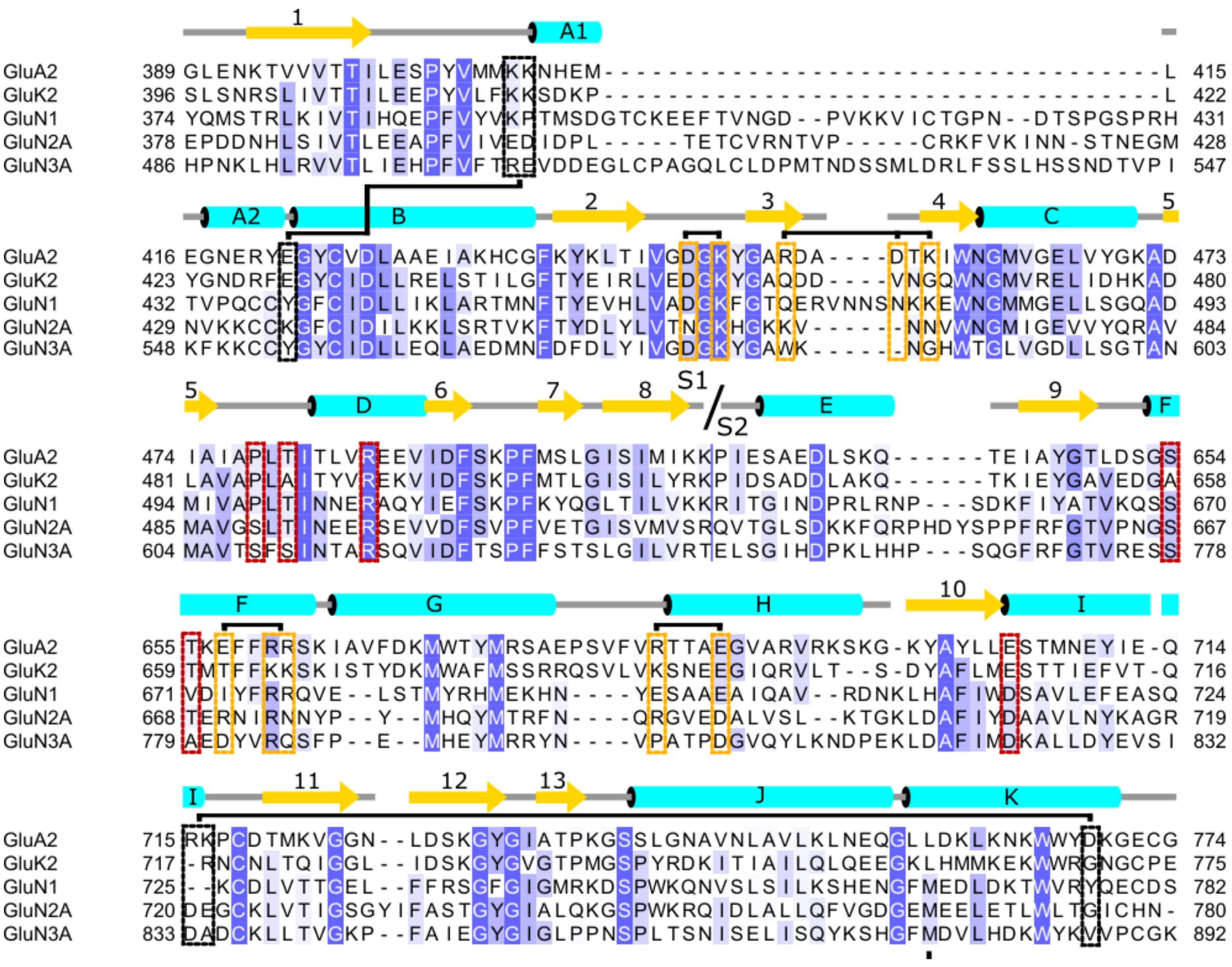

Figure S8. Sequence alignment of iGluR LBDs across AMPA, kainate, and NMDA receptors in rat. Related to Figures 1, 2, and 4. Binding pocket residues are boxed in red. Residues that make metastable interactions are boxed in yellow. Off-pathway residues that were mutated are boxed in black.

Table S1. Ligand-binding trajectories for the dimer and monomer systems. Related to Figures 1, S3, and S4, and Movies S1–S4.

	No. of ligands	Event [a]	Apo, prior [b]	ligand docked [c]	ligand conformation	(ξ1, ξ2) median[d]	Apo, post [e]	Previous Trajectory
T_{dim1}	20	A	0.93	2.49	crystallographic	11.7, 11.1	N/A	N/A
T_{dim2}	20	A	1.39	0.88	crystallographic → inverted	13.1, 11.5	N/A	N/A
T_{dim3}	20	A	N/A	3.12	inverted	12.9, 11.6	N/A	T_{dim6}
T_{dim4}	20	A	0.08	1.4	inverted	12.7, 11.2	N/A	T_{dim5}
T_{dim5}	20	D	N/A	2.49	crystallographic	11.7, 11.1	0.08	T_{dim1}
T_{dim6}	20	D	N/A	0.88	inverted	13.1, 11.5	N/A	T_{dim2}
T_{dim7}	20	D	N/A	1.4	inverted	12.7, 11.2	0.48	T_{dim4}
T_{dim8}	2	D	N/A	1.44	crystallographic	11.5, 11.1	4.98	N/A
T_{dim9}	2	D	N/A	3.57	crystallographic	11.7, 10.8	2.85	N/A
T_{mon1}	1	A	2.94	2.04	inverted	12.6, 11.1	N/A	T_{mon4}
T_{mon2}	10	A	1.25	0.78	inverted	14.1, 11.7	N/A	N/A
T_{mon3}	10	D	N/A	0.78	inverted	14.1, 11.7	0.88	T_{mon2}
T_{mon4}	1	D	N/A	1.25	crystallographic	11.1, 11.4	2.94	N/A
T_{mon5}	1	D	N/A	0.98	crystallographic	11.2, 10.7	9.32	N/A
T_{mon6}	1	D	N/A	5.52	crystallographic → inverted	12.4, 11.7	5.21	N/A
T_{mon7}	1	D	N/A	1.84	crystallographic	11.5, 11.0	7.2	N/A
T_{mon8}	1	D	N/A	1.85	crystallographic	11.8, 11.2	6.51	N/A

[a] Binding events are labeled A for association and D for dissociation
[b] μs in the apo conformation prior to the binding event
[c] μs in the docked ligand conformation
[d] units are in Ångstroms
[e] μs in the apo conformation after the binding event

Table S2. Quantities used in the calculation of k_{on} (= 1.4×10^7 M^{-1} s^{-1}). Related to STAR Methods.

No. of subunits	No. of Ligands	Free Ligand Concentration (mM)	Time spent in bulk solvent (μs)	Binding Events
1	1	3.9	31.18	1
2	1	3.5	2.13	0
2	2	6.9	2.85	0
1	9	35.1	0.78	0
1	10	38.9	2.13	1
2	18	63.5	3.43	2
2	19	67.1	1.02	1
2	20	70.6	0.93	1

Table S3. Kinetic properties of WT GluA2 and mutants. Related to Figures 4–6.

Mutant / Condition	$t_{10\text{-}90\%\ rise}$, μs (*n*)	*P*	τ_{deact}, ms (*n*)	*P*	τ_{des}, ms (*n*)	*P*	τ_{rec}, ms (*n*)	*P*
WT GluA2	190 ± 30 (4)		1.5 ± 0.2 (6)		10.4 ± 1.1 (8)		59 ± 4 (3)	
R453D	610 ± 60 (4)	0.005	3.6 ± 0.1 (3)	0.08	8.4 ± 0.6 (3)	0.2	31 ± 3 (3)	0.01
K458D	330 ± 30 (3)	0.03	1.4 ± 0.1 (3)	0.7	11.7 ± 1.3 (3)	0.5	60 ± 5 (3)	0.8
R453A, D456A, K458A (RDK-AAA)	730 ± 80 (6)	0.0003	3.7 ± 0.1 (4)	0.003	7.1 ± 0.7 (7)	0.03	32 ± 5 (3)	0.02
R660E	250 ± 10 (4)	0.2	2.4 ± 0.2 (4)	0.02	8.6 ± 0.6 (4)	0.2	75 ± 9 (3)	0.3
R661E	300 ± 30 (3)	0.07	3 ± 0.1 (3)	0.07	10.8 ± 2.3 (3)	0.9	48 ± 3 (3)	0.1
E657A, R660A, R661A (ERR-AAA)	550 ± 80 (6)	0.004	3.8 ± 0.4 (4)	0.01	7.8 ± 1.7 (6)	0.2	45 ± 9 (3)	0.3
D447A, K449A (DK-AA)	410 ± 40 (4)	0.01	3.1 ± 0.5 (3)	0.1	6.8 ± 0.8 (4)	0.03	40 ± 5 (3)	0.06
R684A, E688A (RE-AA)	590 ± 50 (5)	0.0002	4.9 ± 0.7 (3)	0.04	10.8 ± 1.3 (5)	0.8	13 ± 1 (3)	0.008
K409A, K410A, E422A (KKE-AAA)	310 ± 10 (3)	0.04	1.1 ± 0.1 (4)	0.1	11.1 ± 1.3 (4)	0.7	53 ± 8 (3)	0.6
R715A K716A D769A (RKD-AAA)	100 ± 5 (4)	0.07	2.4 ± 0.1 (4)	0.005	9 ± 0.7 (4)	0.4	28 ± 4 (3)	0.01
WT GluA2 (50 mM Glu)	180 ± 40 (3)		1.2 ± 0.1 (3)		6.6 ± 0.8 (3)			
RDK-AAA (50 mM Glu)	660 ± 70 (3)	0.01	3.7 ± 0.1 (3)	0.0003	7.7 ± 0.3 (3)	0.3		

All experiments were done with 10 mM glutamate except where noted. Activation and deactivation data are plotted in Figures 4–6. The number of experiments is indicated in brackets. $t_{10\text{-}90\%\ rise}$, rise time; τ_{deact}, time constant from single exponential fits to the deactivation decay; τ_{des}, time constant from single exponential fits to the desensitization decay; τ_{rec}, recovery time constant from Hodgkin-Huxley fits with $h = 2$ (see STAR Methods). *P* values are from two-tailed Student's *t*-test against WT values.

Table S4. Ligand-binding trajectories for the ERR-AAA mutant LBD. Related to Figure 5 and Movie S4.

	No. of ligands	Event [a]	Apo, prior [b]	ligand docked [c]	ligand conformation	(ξ1, ξ2) median[d]	Apo, post [e]	Previous Trajectory
T_{mut1}	10	A	0.06	0.31	inverted	15.3, 11.8	N/A	N/A
T_{mut2}	10	A	0.10	0.75	inverted	12.8, 11.5	N/A	T_{mut3}
T_{mut3}	10	D	N/A	0.31	inverted	15.3, 11.8	0.10	T_{mut1}
T_{mut4}	10	D	N/A	0.75	inverted	12.8, 11.5	N/A	N/A

[a] Binding events are labeled A for association and D for dissociation
[b] μs in the apo conformation prior to the binding event
[c] μs in the docked ligand conformation
[d] units are in Ångstroms
[e] μs in the apo conformation after the binding event

Movie S1. Glutamate binding in the crystallographic conformation. Related to Figure 1. The binding events shown correspond to T_{dim1}, a system consisting of the LBD dimer with 20 ligands. For clarity, the opposing subunit and ligands that do not bind are not shown.

Movie S2. Glutamate binding in the inverted conformation. Related to Figure 3 and Figure S3. The binding events shown correspond to Figure S3 and T_{mon1} in a system consisting of the LBD monomer with one ligand.

Movie S3. Glutamate converting from the crystallographic to inverted conformations within the binding pocket. Related to Figure 3 and Figure S4. The binding events shown correspond to T_{dim2}, a system consisting of the LBD dimer with 20 ligands. For clarity, the opposing subunit and ligand that do not bind are not shown.

Movie S4. Glutamate binding in the ERR-AAA mutant LBD. Related to Figure 5. The binding events shown correspond to T_{mut1}, a system consisting of the LBD monomer with 10 ligands. This movie starts with the ligand's α- and γ-carboxylates interacting with R684 and R675, respectively, on Lobe 2. Prior to this interaction, the ligand was diffusing in bulk solvent. Interactions between the ligand and Lobe 2 residues break, and the ligand diffuses into the binding pocket to contact R485. R485 initially coordinates the ligand's α-carboxyl group, but these contacts are subsequently severed, and the ligand rotates such that R485 coordinates the γ-carboxyl group. The

ligand then transitions into the binding pocket and adopts the inverted pose. Interactions are formed between the ligand's amide nitrogen and Y450 and E705. For clarity, the ligands that do not bind are not shown.
