# Supplementary material for: Neurotransmitter Funneling Optimizes Glutamate Receptor Kinetics
Source: Neuron. 2018 Jan 3;97(1):139–149.e4. doi: 10.1016/j.neuron.2017.11.024 (PMC5766834; doi:10.1016/j.neuron.2017.11.024)
Supplement: Document S2. Article plus Supplemental Information [file mmc6.pdf]

Article

Neuron

Neurotransmitter Funneling Optimizes Glutamate Receptor Kinetics

Highlights

- Glutamate binds to AMPA receptors by following preferential pathways
- All-atom simulations find metastable sites that speed binding and unbinding
- Perturbing glutamate-binding pathways slows both activation and deactivation
- Glutamate can bind in two orientations in its pocket

Authors

Alvin Yu, Héctor Salazar,
Andrew J.R. Plested, Albert Y. Lau

Correspondence

plested@fmp-berlin.de (A.J.R.P.),
alau@jhmi.edu (A.Y.L.)

In Brief

Yu et al. show that the ligand-binding domain of ionotropic glutamate receptors has structural features on its surface that guide the agonist glutamate to its binding site. This phenomenon serves to accelerate receptor activation.

Yu et al., 2018, Neuron *97*, 139–149
January 3, 2018 © 2017 The Authors. Published by Elsevier Inc.
https://doi.org/10.1016/j.neuron.2017.11.024

CellPress

CellPress

Neurotransmitter Funneling Optimizes Glutamate Receptor Kinetics

Alvin Yu,[1,2,6] Héctor Salazar,[3,4,5,6] Andrew J.R. Plested,[3,4,5,*] and Albert Y. Lau[1,2,7,*]
[1]Program in Molecular Biophysics, Johns Hopkins University, Baltimore, MD 21218, USA
[2]Department of Biophysics and Biophysical Chemistry, Johns Hopkins University School of Medicine, Baltimore, MD 21205, USA
[3]Leibniz-Forschungsinstitut für Molekulare Pharmakologie, 13125 Berlin, Germany
[4]Cluster of Excellence NeuroCure, Charité-Universitätsmedizin Berlin, 10117 Berlin, Germany
[5]Institute of Biology, Cellular Biophysics, Humboldt Universität zu Berlin, 10115 Berlin, Germany
[6]These authors contributed equally
[7]Lead Contact
*Correspondence: plested@fmp-berlin.de (A.J.R.P.), alau@jhmi.edu (A.Y.L.)
https://doi.org/10.1016/j.neuron.2017.11.024

SUMMARY

Ionotropic glutamate receptors (iGluRs) mediate neurotransmission at the majority of excitatory synapses in the brain. Little is known, however, about how glutamate reaches the recessed binding pocket in iGluR ligand-binding domains (LBDs). Here we report the process of glutamate binding to a prototypical iGluR, GluA2, in atomistic detail using unbiased molecular simulations. Charged residues on the LBD surface form pathways that facilitate glutamate binding by effectively reducing a three-dimensional diffusion process to a spatially constrained, two-dimensional one. Free energy calculations identify residues that metastably bind glutamate and help guide it into the binding pocket. These simulations also reveal that glutamate can bind in an inverted conformation and also reorient while in its pocket. Electrophysiological recordings demonstrate that eliminating these transient binding sites slows activation and deactivation, consistent with slower glutamate binding and unbinding. These results suggest that binding pathways have evolved to optimize rapid responses of AMPA-type iGluRs at synapses.

INTRODUCTION

The speed of information processing in the brain is limited to a maximum rate of ~1 kHz by the width of action potentials, the release rate of synaptic vesicles, and the duration of synaptic potentials (Attwell and Gibb, 2005; Lisman et al., 2007). Fast activation and deactivation of synaptic neurotransmitter receptors is therefore essential for normal signaling in the nervous system. One of the fastest-operating receptors is the AMPA-type ionotropic glutamate receptor (iGluR) (Baranovic and Plested, 2016), a ligand-gated ion channel found throughout the brain (Traynelis et al., 2010). Activation of these receptors is the final step in a cascade that allows cells releasing glutamate to excite downstream target neurons with millisecond precision (Mayer, 2011). Each receptor has four binding sites for glutamate that resemble clamshells, termed ligand-binding domains (LBDs). The four LBDs in each receptor assemble as a dimer of dimers (Mayer, 2016). Because glutamate-bound LBDs are closed, ligand binding is thought to pull open the gate of the attached ion channel pore. Alternatively, binding can drive separate conformational changes that result in an unresponsive, desensitized receptor (Sun et al., 2002). A paradox of AMPA receptor activation is how the dynamics of both binding and unbinding of glutamate are fast enough in order to allow both the onset and termination of activity to be rapid, yet robust and selective.

Over 100 high-resolution structures of genetically isolated iGluR LBDs have been determined, in complex with agonists and competitive antagonists, as well as in the apo state. These structures reveal common modes of ligand binding within the cleft (Mayer, 2016). Computational analyses, in turn, have shed light on both LBD and ligand dynamics and energetics (Arinaminpathy et al., 2002; Dai and Zhou, 2016; Lau and Roux, 2007, 2011; Mamonova et al., 2008; Mendieta et al., 2005; Okada et al., 2012; Postila et al., 2010; Sahai and Biggin, 2011; Speranskiy and Kurnikova, 2005; Wolter et al., 2013; Yao et al., 2013). Neither the structural nor computational studies, however, have comprehensively shown how glutamate finds its way into the binding site or how large-scale conformational changes in the LBD are coupled to glutamate binding. For example, does glutamate diffuse directly from bulk solvent into its binding pocket, only contacting the protein randomly, or does glutamate follow distinct pathways on the surface of the LBD to find its way into its pocket? If the latter, what structural features make up the pathways, and what is the nature of the protein-ligand interactions therein? In this study, long-timescale molecular dynamics (MD) simulations (totaling ~50 μs) and free energy calculations of the GluA2 receptor suggest that strategically positioned flexible side chains on the surface of the LBD metastably interact with glutamate to help guide, or "funnel," it into its recessed binding pocket, where it adopts two possible poses. These results were used to guide the design of a panel

This is an open access article under the CC BY license (http://creativecommons.org/licenses/by/4.0/).

of LBD mutants that were tested using electrophysiological recordings. Elimination of the transient binding sites was found to slow both activation and deactivation of the receptor. Taken together, these results suggest that glutamate, and perhaps other iGluR ligands as well, binds via distinct pathways on the surface of the LBD, and disruption of these pathways significantly impacts the functional kinetics of the receptor.

RESULTS

Glutamate Binds via Preferred Pathways and Metastable Interactions

In order to examine the processes by which a glutamate ligand either associates with or dissociates from the GluA2 AMPA receptor LBD, we performed unbiased all-atom MD simulations with explicit solvent using special-purpose hardware (Shaw et al., 2009) to generate 21 trajectories (17 trajectories involved the wild-type [WT] LBD, and 4 trajectories involved an LBD variant) with an aggregate simulation time of 49.1 μs. The four LBDs of an iGluR are arranged as a dimer of dimers; we simulated glutamate binding in both LBD dimers and monomers. The binding of glutamate is thought to involve the following: ligand entry into an open, solvent-exposed binding cleft; ligand docking to R485 in Lobe 1 of the LBD; then large-scale conformational changes that close the cleft, securing the ligand (Cheng et al., 2005). After glutamate has docked, cleft closure proceeds when the ligand, attached to R485, also attaches to E705 in Lobe 2 (Armstrong and Gouaux, 2000). The ligand forms both of these Lobe 1 and Lobe 2 interactions in all of the simulated binding trajectories, with the caveat that, in some of the simulations, the final orientation of glutamate was not the same as that observed in crystallographic studies (see below).

In our simulation systems (see Tables S1 and S2), glutamate ligand molecules were initially placed at random positions and orientations in bulk solvent, similar to the approach of Dror et al. (2011), at least 8 Å away from any non-water molecules. The effective ligand concentrations ranged from 3.9 mM (single ligand in a monomer system) to 71 mM (20 ligands in a dimer system). These concentrations are somewhat higher than estimates of the peak glutamate concentration during synaptic transmission (1–10 mM) (Clements et al., 1992; Rudolph et al., 2015), but our goal was to maximize the number of independent binding events that we could obtain with our allocation of computational resources. Despite the high concentrations, we did not observe any artifactual ligand-ligand or protein-ligand interactions. In all but one of the binding trajectories, the carboxyl groups of glutamate were exploited by positively charged side chains at the periphery of the binding cleft. Strikingly, glutamate was passed from one residue to the next by a select set of residues that funnel the neurotransmitter, via a series of metastable interactions, into its binding site.

In one such trajectory, the ligand first contacts the LBD at R453 on loop 2 (Figure 1; Movie S1). From there, its γ-carboxyl group swings toward Lobe 2, interacting with helix F residues E657, R660, and R661. Mutations in this region, a prominent metastable interaction site (occurring in three out of six binding simulations), alter AMPA receptor function (Weston et al., 2006). Unexpectedly, the guanidinium group of R485 in helix D rotates dramatically out of the binding pocket to contact the ligand. This surprising conformational flexibility occurs via rotations of –106°, –127°, –136°, and –111° around the R485 side chain's χ_1, χ_2, χ_3, and χ_4 torsion angles, respectively (Figures 1E–1I). The ligand remains tethered to R485 as it leaves the metastable site, being pulled into the binding cleft. Finally, the ligand's amine contacts E705 to close the cleft and secure the ligand in the crystallographic pose. Water molecules were observed to occupy approximately the same positions in the cleft as those seen in crystal structures (Armstrong and Gouaux, 2000). Prior to ligand binding, a water molecule (W1) contacts the E705 amine, and another (W2) mediates a hydrogen bond interaction between the L650 amine and the L703 backbone carbonyl. Contact between the ligand's amine and E705 reduces the exchange frequency of water molecules in the cleft with bulk solvent. One water molecule (W3) is recruited during ligand binding to the base of helix F after the ligand's amine contacts the E705 side chain. In our binding trajectories, the LBD does not close as fully as is seen crystallographically ((ξ_1, ξ_2) = (11.8, 10.8 Å) versus (ξ_1, ξ_2) = (9.5, 7.8 Å) for PDB: 1FTJ; see Discussion below). Comparison with the crystal structure reveals the ligand-bound complex lacks a γ-carboxyl group interaction with the backbone amine of T655 in Lobe 2. Details of individual trajectories are provided in Table S1.

The binding pathways in the dimer and monomer simulations are very similar. Given the functional independence of binding processes and the location of the pathways at the periphery of the LBD tetramer (Figure S1), we expect that the pathways we observe are the same in full-length, tetrameric receptors. In all trajectories, K730, in the hinge region between Lobes 1 and 2, shows substantial conformational flexibility and alternates between forming salt bridge interactions with E705 in Lobe 2 and D728 in the hinge. From our simulations, it is unclear how much these interactions contribute to "locking" the LBD closed, as proposed by Armstrong and Gouaux (2000).

Potential of Mean Force for Binding and Unbinding

To understand the energetics of funneling in each of the pathways, we computed a three-dimensional free energy landscape, or potential of mean force (PMF), from our binding and unbinding trajectories. The PMF, shown in Figure 2A, indicates three possible ligand-binding pathways. Contouring the PMF at lower energies reveals sites of metastable protein-ligand interactions (Figures 2B–2D). Situated inside the binding pocket, site 0 is the global free energy minimum, set to 0 kcal/mol. The next most stable sites, sites 1, 2, and 3, are local minima with free energies of 0.29, 0.83, and 0.75 kcal/mol, respectively, located at positions where the ligand forms metastable interactions with the LBD. Ligand positions in bulk solvent, on the other hand, have free energies of about 3.12 kcal/mol. The ligand traverses Pathway 1 in about half of the trajectories, pausing at site 1 prior to binding at site 0. In Pathway 2, the ligand interacts with sites 2 and 1 prior to binding. In Pathway 3, the ligand transitions from site 3 directly to site 0. Residue-ligand interactions play a role in passing the ligand from each site to the next. In particular, helix F residues (E657, R660, and R661) are involved in interactions at sites 1 and 2 as well as transitions from site 2 to site 1 and from site 1 to site 0. K449 is involved in shuttling the ligand into the

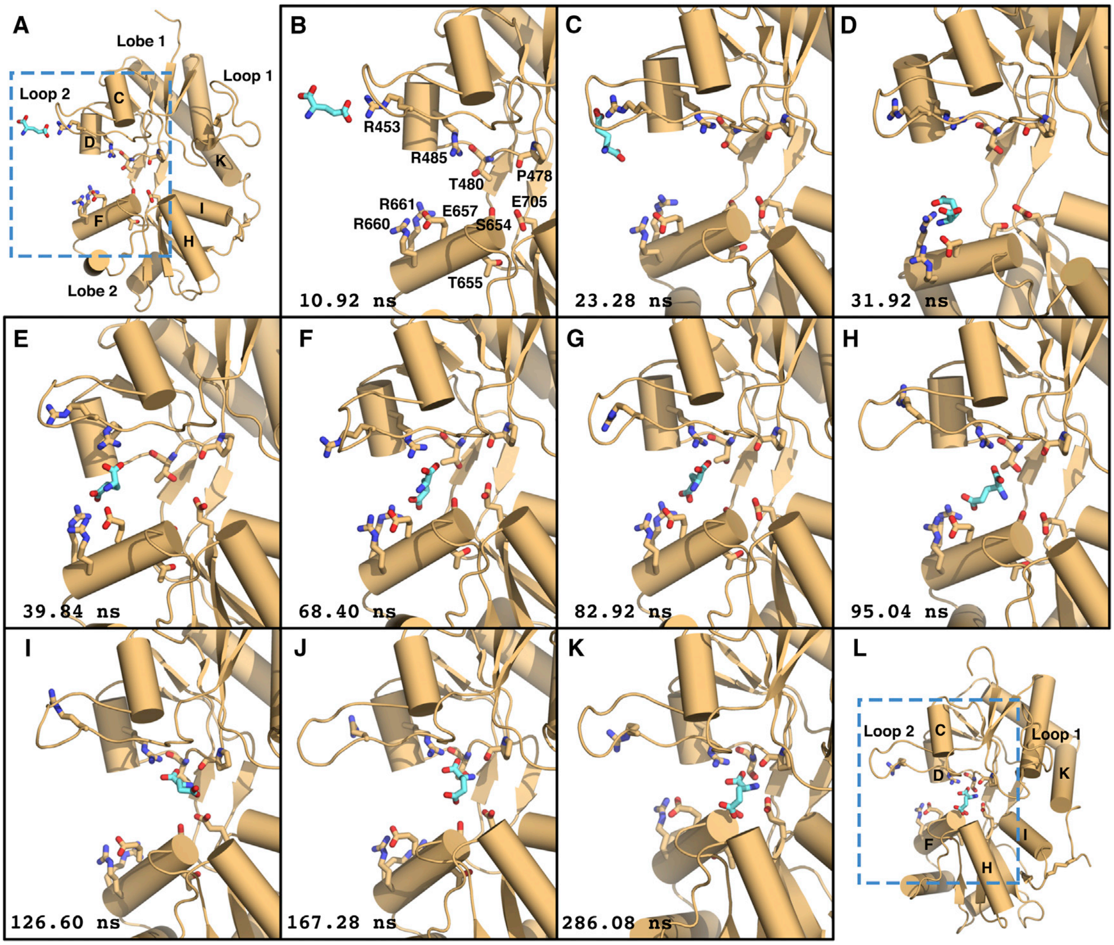

Figure 1. Dynamics of Glutamate Binding

Time points (lower left of each panel) are relative to the start of Movie S1. This trajectory corresponds to the LBD dimer system T_{dim1} (see Table S1); only the LBD that binds glutamate is shown for clarity.

(A) Prior to ligand entry to the binding pocket, the LBD is open; (ξ_1, ξ_2) = (12.3, 12.2 Å). The ligand's γ-carboxylate contacts R453 on Lobe 1.

(B) Close-up view of (A).

(C) Glutamate slips into the binding cleft.

(D) The ligand contacts R661 on Lobe 2.

(E) A metastable interaction forms across Lobes 1 and 2. The ligand's γ-carboxylate contacts E657, R660, and R661 on helix F, and the ligand's α-carboxylate contacts R485. R485 flickers out of the binding pocket to interact with the ligand.

(F) R485 relaxes toward the binding pocket.

(G) The metastable interaction at the ligand's α- and γ-carboxylate between Lobes 1 and 2 persists.

(H) The ligand's α-carboxylate and amine move to interact with binding pocket residues in Lobe 1.

(I) The ligand shifts into the binding pocket, with its α-carboxylate contacting R485. Lobe 2 interactions with helix F are broken. In the pocket, the ligand's amine is coordinated by P478 and L480. Cleft closure is initiated once helix F undergoes a backward tilt to form a pocket for the ligand's γ-carboxylate.

(J) Glutamate adopts the crystallographic conformation.

(K) As the cleft closes to secure the ligand, the ligand's amine contacts E705 on Lobe 2, and the ligand's γ-carboxylate contacts the backbone amine of S654.

(L) Expanded view of (K). The LBD closes around the ligand in the crystallographic conformation: (ξ_1, ξ_2) = (11.8, 10.8 Å).

See also Figures S1 and S8, Movie S1, and Table S1.

binding pocket from site 3. A list of the residues that interact with the ligand at each site is provided in the legend for Figure 2A. An error analysis of the PMF is provided in Figure S2.

The association rate constant calculated from our simulations, k_{on} = 1.4 × 10^7 M^{-1} s^{-1}, using the approach of Dror et al. (2011) (see STAR Methods and Table S2), agrees closely with the experimentally measured value of 1.6 × 10^7 M^{-1} s^{-1} for the GluA4 LBD (Abele et al., 2000). To our knowledge, no experimentally measured k_{on} for the GluA2 LBD has been reported, but the sub-millisecond activation lag at 10 mM glutamate reported here and elsewhere is consistent with this value.

Glutamate May Adopt an Inverted Pose during Binding

The lack of preference for binding the α- or γ-carboxylates of glutamate at metastable sites presents a paradox. How is glutamate delivered efficiently into the pose seen in crystallographic

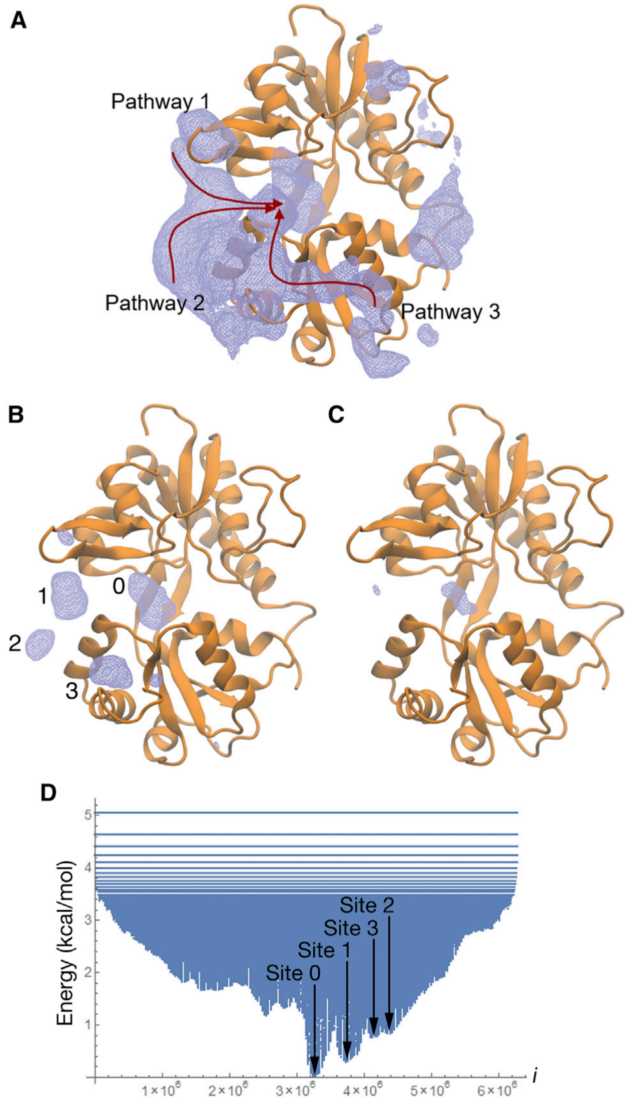

Figure 2. Glutamate Binding Pathways and Metastable Binding Sites

(A) The PMF calculated from the ligand density using a hard-sphere van der Waals approximation on a grid spacing of 0.5 Å along the x, y, and z axes contoured at 1.89 kcal mol^{-1}. Data are from the monomeric LBD system with 10 ligands ($T_{mon2,3}$). The primary binding pathways for glutamate are depicted by arrows.

(B) The PMF, contoured at 1.16 kcal mol^{-1}, defines the regions of metastable interaction, sites 0–3. Site 0 is the site of stable binding. Site 1 is shared by Pathways 1 and 2, site 2 is encountered in Pathway 2, and site 3 is encountered in Pathway 3. Site 1 spans the two lobes, involving R453 in Lobe 1 and E657, R660, and R661 in Lobe 2. Site 2 involves E657, R660, and R661 in Lobe 2. Site 3 involves R675 and R684 in Lobe 2. The residues involved in site 0 are shown in Figure 3A. Error analysis is provided in Figure S2.

(C) The PMF, contoured at 0.32 kcal mol^{-1}, shows the global free energy minimum. This minimum overlaps well with the ligand density derived from crystal structures of the glutamate-bound complex (e.g., PDB: 1FTJ).

(D) The one-dimensional representation of the WT GluA2 ligand-binding PMF was obtained by first computing the three-dimensional PMF (A–C). Values of the three-dimensional PMF were indexed increasing in the *z*, *y*, then *x* directions, from (x_{min}, y_{min}, z_{min}), to produce the one-dimensional representation. The positions of sites 0–3 are indicated. Site 0 is the global free energy minimum and is set to 0 kcal/mol; sites 1–3 form local minima with free energies of 0.29, 0.83, and 0.75 kcal/mol, respectively.

See also Figures S2 and S8.

experiments? Two of the association trajectories resulted in glutamate binding into the crystallographically observed pose (Armstrong and Gouaux, 2000), in which the ligand's α-carboxyl group is anchored by R485 in Lobe 1 while the γ-carboxyl group is stabilized by the backbone amine of S654 in Lobe 2 (Figure 3A). The other four trajectories unexpectedly resulted in glutamate binding into an "inverted" pose, in which the γ-carboxyl group binds to Lobe 1 and the α-carboxyl group binds to Lobe 2 (Figure 3B; Figure S3; Movie S2). However, in one simulation (Figure S4; Movie S3; T_{dim2} in Table S1), glutamate rotates from the crystallographic pose to the inverted pose, suggesting that interconversion between poses is possible. In this case, the binding cleft was not fully closed. Free energy landscapes governing cleft closure have been described using the two-dimensional order parameter (ξ_1, ξ_2) (Figure 3C) (Lau and Roux, 2007, 2011; Yao et al., 2013; Yu et al., 2016). The extent of cleft closure with glutamate bound in the inverted pose is not as great as that seen in crystal structures or in simulations with the ligand in the crystallographic pose (Figure 3D). It is possible that an extension of T_{dim2} could have resulted in the ligand returning to the crystallographic pose. In support of this notion, MD simulations of drug binding to G-protein-coupled receptors showed that a drug, initially bound in a non-crystallographic pose, eventually converted to the crystallographic pose (Dror et al., 2011). Our measurements do not indicate how stable the non-crystallographic, inverted pose is, but this binding mode could cause glutamate to act as a partial agonist with a smaller degree of cleft closure. Individual AMPA receptor activations show substantial sublevel activity, connected to LBD occupancy (Rosenmund et al., 1998). Stochastic fluctuations in the single channel current could additionally reflect dynamic conversions between crystallographic and non-crystallographic ligand poses. Experimental data are not yet available to confirm the physiological relevance of the non-crystallographic binding pose detected in our simulations. Additional biophysical studies are needed to understand how it might contribute to iGluR function.

Unbinding Pathways Mirror Binding Pathways

We also simulated ligand dissociation trajectories, which we initiated from either crystal structure-like configurations in which the LBD is fully closed with glutamate in the crystallographic pose (PDB: 1FTJ) or ligand-docked configurations in which the LBD is semi-closed or open (Table S1). The latter trajectories were continuations of prior ligand-association trajectories. As with the association trajectories, all dissociation trajectories occurred spontaneously. In general, for both the LBD dimer and monomer systems, binding and unbinding pathways appear to be the reverse of each other, with free energy barriers being traversed in opposite order. The ligand exited from the ξ_1 side of the binding cleft in all five dissociation events involving the dimer. For the monomer, the ligand exited from the ξ_1 side in four dissociation events and from the ξ_2 side in two events. In one instance, a ligand that dissociated from the LBD in the crystallographic pose ended up rebinding in the inverted pose (T_{mon4} to T_{mon1}, Table S1).

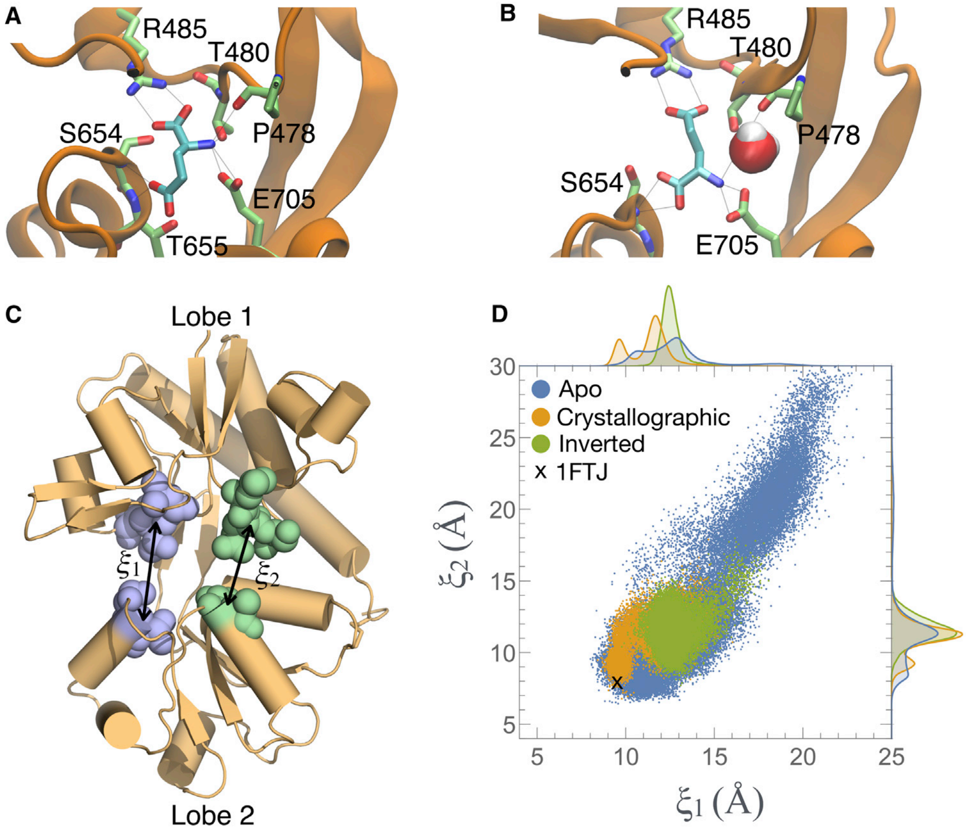

Figure 3. Conformations of Bound Glutamate and the LBD

(A) The bound ligand conformation, taken from PDB: 1FTJ. The ligand's α-carboxylate contacts R485, and its γ-carboxylate contacts the backbone amine of S654. The ligand's amine is coordinated to the side chains of T480 and E705 and to the backbone of P478.

(B) The inverted conformation of the ligand. The ligand's α-carboxylate contacts the backbone amine of S654, and its γ-carboxylate contacts R485. The ligand's amine is coordinated to E705. P478 has moved upward to accommodate a water molecule that also contacts the ligand's amine.

(C) The two-dimensional order parameter (ξ_1, ξ_2) used to characterize large-scale conformational transitions in the GluA2 LBD. ξ_1 and ξ_2 each indicate the distance between the centers of mass of the clusters of atoms shown in blue and green, respectively.

(D) (ξ_1, ξ_2) measures the degree of cleft closure for the LBD in apo (blue) and ligand-bound conformations. The ligand occupies either the crystallographic (orange) or the inverted (green) poses. Each point represents a snapshot taken every 120 ps from simulations of the monomer system at 3.9 mM glutamate concentration ($T_{mon1,4-8}$). The marginal histograms indicate distribution densities. The "X" indicates the degree of cleft closure in the crystal structure (PDB: 1FTJ).

See also Figures S3 and S4 and Movies S2 and S3.

Disrupting Binding Pathways Selectively Slows Both Activation and Deactivation

The simulations suggest that clusters of charged residues along the binding pathways should interact with glutamate during both association and dissociation. To test this hypothesis, we designed a set of single, double, and triple mutants, removing or reversing the polarity of charged side chains at each metastable site in turn (Figure 4A). We hypothesized that these mutations should alter receptor activation, deactivation, and perhaps recovery from desensitization. Given that the metastable sites are formed by flexible residues lacking interactions with other parts of the receptor, we expected little effect of the mutations on downstream gating conformational changes. Additionally, we expected that entry into desensitization, which likely involves neither binding nor conformational changes of the individual LBDs, should be unaffected.

Association is the first step in the transition from resting to activated states. Therefore, we measured receptor activation by 10 mM glutamate in outside-out patches as a proxy for glutamate association. Anticipating that the mutations could slow activation, we used long pulses (200 ms) to ensure that the peak of the response was reached. This approach conveniently allowed us to measure the rate of desensitization from the same records (see below). Desensitization had minimal effect on the activation time, being ~50-fold slower. Activation of WT GluA2 by 10 mM glutamate is very fast, with a 10%–90% rise time of 190 ± 30 μs. The rise time is probably slowed by the rate of solution exchange onto the excised patch. Strikingly, a substantial 3-fold increase in the 10%–90% rise time during activation, t_{rise}, was observed for R453D and a triple-alanine mutant, R453A-D456A-K458A (denoted RDK-AAA; t_{rise} = 610 ± 60 and 730 ± 80 μs, respectively; $p < 0.005$ versus WT GluA2, t test, n = 3–7; Figures 4B and 4C; Table S3).

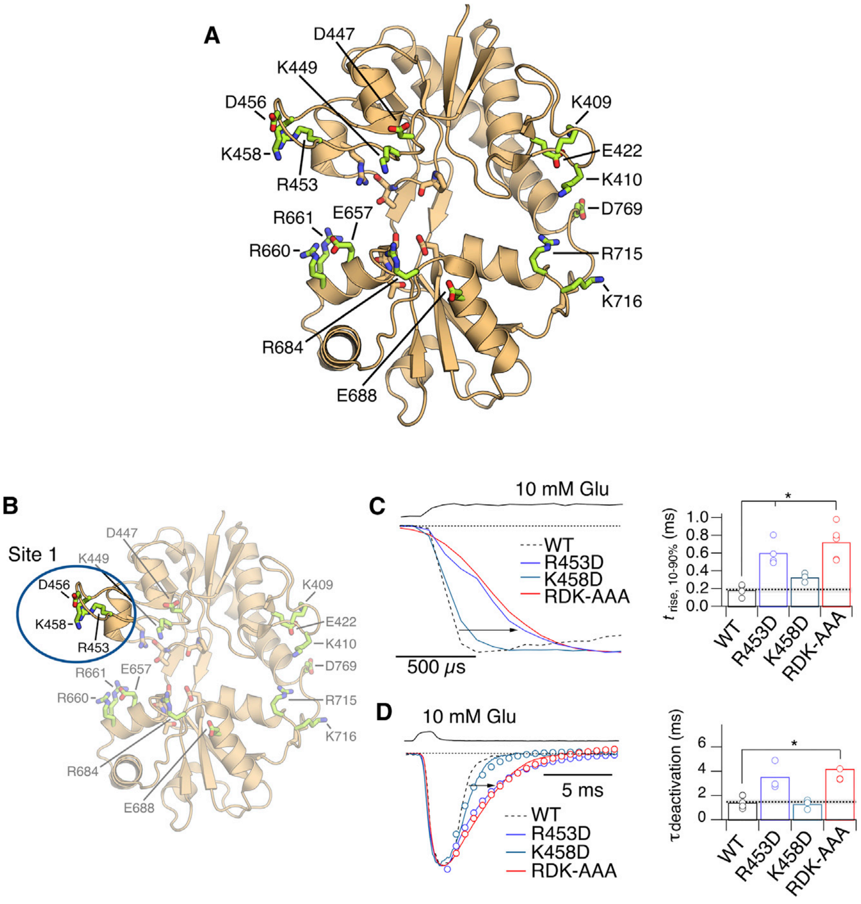

Figure 4. Activation and Deactivation of Receptors with Mutations in Pathway 1

(A) Sites of mutations that were tested functionally. The green-colored side chains correspond to the WT residues. The tan-colored residues contact bound glutamate directly but were not mutated in the functional tests.

(B) The blue oval indicates LBD residues proximal to metastable site 1 (Figure 2B) in Lobe 1. R453 interacts with the ligand in the simulations. Mutants tested include single-charge swaps R453D and K458D, and the triple mutant R453A-D456A-K458A (RDK-AAA).

(C) Activation of Lobe 1 mutants (R453D, blue; K458D, green; RDK-AAA, red) by a long pulse of 10 mM glutamate. Solution exchange measured after the experiment is shown as the upper black trace. A typical WT GluA2 response is plotted with a dotted line. The individual 10%–90% rise times of the currents (t_{rise}) are shown in the bar chart in the right panel, with the WT mean value as a dashed gray line. Asterisk indicates $p < 0.005$, Student's t test.

(D) Left panel shows deactivation of Lobe 1 mutants in response to ~1 ms pulse of 10 mM glutamate. Color coding is as in (C), with monoexponential fits indicated by open circles. Right panel shows bar chart of individual deactivation decay values. Asterisk indicates $p < 0.005$, Student's t test.

See also Figures S1, S5, S6, and S8 and Table S3.

We next examined receptor deactivation, a process limited by the glutamate unbinding rate, by applying short (1 ms) pulses of 10 mM glutamate. WT GluA2 deactivates rapidly, with a deactivation time constant, τ_{deact}, of 1.5 ± 0.2 ms. Mutating residues involved in metastable site 1, we observed a robust increase in τ_{deact} for R453D (τ_{deact} = 3.6 ± 0.1 ms; Table S3) and RDK-AAA (τ_{deact} = 3.7 ± 0.1 ms), compared with WT GluA2 (p = 0.003, t test, n = 3–5) (Figure 4D).

Single substitutions on helix F (R660 and R661; sites 1 and 2) had little effect on activation or deactivation, but the triple mutant E657A-R660A-R661A (denoted ERR-AAA, Figure 5A) exhibited the same profile of slower activation (t_{rise} = 550 ± 80 μs; Figure 5B; Table S3) and deactivation (τ_{deact} = 3.8 ± 0.4 ms; Figure 5C) as the RDK-AAA mutant. A comparison of EC_{50} values for WT GluA2 (EC_{50} = 330 ± 90 μM) and ERR-AAA (EC_{50} = 410 ± 30 μM) revealed that the apparent affinity of the ERR-AAA mutant for glutamate is similar to that of WT (p = 0.5; t test, n = 3) (Figure 5D). Unfortunately, the poor expression of the RDK-AAA mutant precluded a concentration-response analysis for this mutant. Nonetheless, this result is expected if mutations remove metastable interactions but do not affect the initial or final conformational states, revealing a distinct mechanism to previous reports of LBD mutations that change binding (Robert et al., 2005; Weston et al., 2006).

We also tested the RDK-AAA mutant with 50 mM glutamate to ensure that ligand diffusion was not a factor in slowed activation. Both activation and deactivation remained substantially slower than in WT GluA2 with 50 mM glutamate (t_{rise} = 660 ± 70 μs versus 180 ± 40 μs for WT GluA2; p = 0.01 versus WT GluA2, t test, n = 3; τ_{deact} = 3.7 ± 0.1 ms versus 1.2 ± 0.1 ms for WT GluA2; p = 0.0003 versus WT GluA2, t test, n = 3; Figures S5A and S5B; Table S3). This result strongly suggests that the binding kinetics are principally altered by mutations at metastable sites.

In order to determine how the ERR-AAA mutant perturbs glutamate binding, we performed unbiased simulations involving this mutant LBD (Table S4; Movie S4). PMF calculations indicate that this LBD lacks metastable interactions along Pathways 1 and 2, proximal to helix F (Figure 5E). The dominant binding pathway, which does not involve helix F interactions, is similar to Pathway 3 in the WT LBD.

As expected for mutations that perturb only binding kinetics, desensitization was weakly perturbed by mutations to the ligand-binding pathways. The time constant of entry to desensitization, τ_{desen}, for RDK-AAA, ERR-AAA, and single-point mutants were similar to WT GluA2 (Figures S5C, S6A, and S6B; Table S3). For most mutants, the time constant of recovery from desensitization was similar to WT GluA2 (59 ± 4 ms), but the R453D and RDK-AAA mutants recovered about twice as fast (p = 0.01 and p = 0.02, t test, n = 3; Figures S6C and S6D). These results support the idea that entry to and recovery from desensitization occur with the clamshell closed and glutamate stably bound, even though glutamate must eventually unbind during recovery.

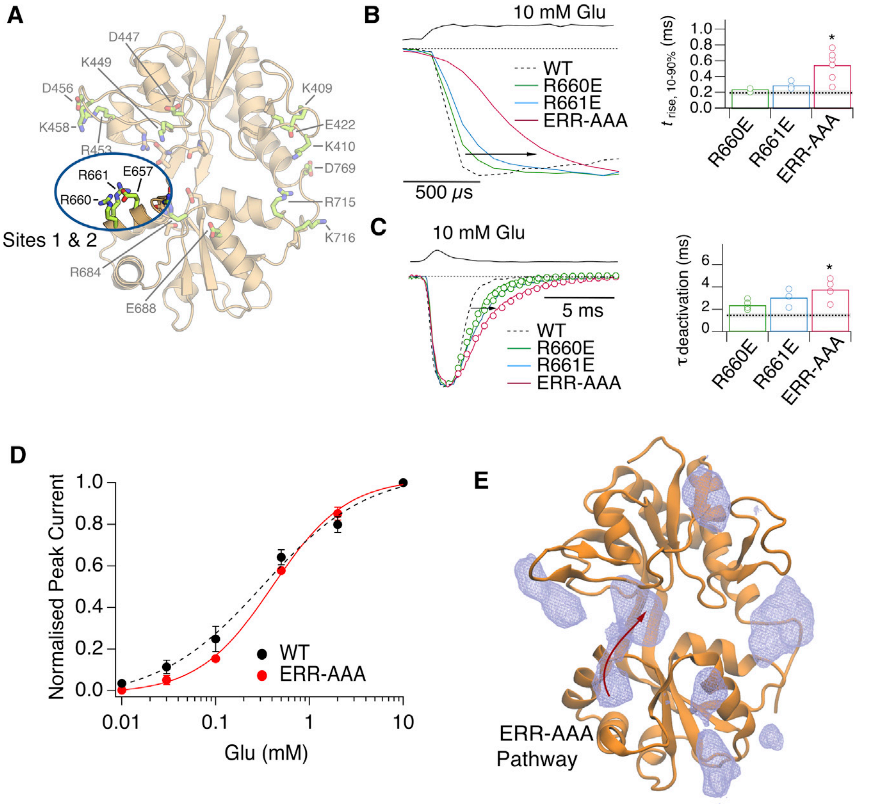

Figure 5. Activation and Deactivation of Receptors with Mutations in Pathways 1 and 2

(A) Residues in Lobe 2 that interact with the ligand at metastable sites 1 and 2 (Figure 2B) include R660, R661, and E657 (blue oval). The following mutations were made to test these sites: R660E, R661E, and E657A-R660A-R661A (ERR-AAA).

(B) Left panel shows activation of Lobe 2 mutants (R660E, green; R661, blue; ERR-AAA, red) in response to a long pulse of 10 mM glutamate. The individual 10%–90% rise times of the currents (t_{rise}) are shown in the bar chart in the right panel, with the WT mean value as a dashed gray line, with asterisk indicating $p < 0.05$ versus WT from t test.

(C) Left panel shows deactivation of Lobe 2 mutants following a 1 ms pulse of 10 mM glutamate, with color coding as in (B). Individual rise times are plotted in the right panel.

(D) The affinity for glutamate is unchanged for the ERR-AAA mutant relative to WT. Dose-response curves in glutamate, measured at the peak current response, for WT GluA2 (EC_{50} = 330 ± 90 μM; black circles), and for the mutant ERR-AAA (EC_{50} = 410 ± 30 μM, red circles). By comparing the fits to responses from individual cells, the glutamate EC_{50} for ERR-AAA was indistinguishable from that of WT GluA2 (p = 0.5; t test, n = 3).

(E) The PMF for the ERR-AAA mutant, contoured at 2.62 kcal/mol. Data are from the monomeric LBD with 10 ligands (T_{mut1-4}). The PMF shows a loss of ligand density along Pathways 1 and 2, proximal to helix F. The ERR-AAA ligand-binding pathway, indicated by the red arrow, resembles Pathway 3 of the WT LBD. Interactions between R684 and R675 on Lobe 2 at site 4 are preserved in both the mutant and WT protein. These residues metastably interact with the ligand prior to binding in T_{mut1}.

See also Figure S6, Tables S3 and S4, and Movie S4.

The second set of mutants that we generated targeted Pathway 3. The D447A-K449A double mutant in Lobe 1 (DK-AA) slowed activation (t_{rise} = 410 ± 40 μs; p = 0.01, versus WT GluA2, t test, n = 4–6; Figures 6A and 6B; Table S3) compared with WT GluA2. The very poor expression of this mutant precluded a robust measurement of deactivation (τ_{deact} = 3.1 ± 0.5 ms; p = 0.1 versus WT GluA2, t test, n = 3; Figure 6C). The R684A-E688A double mutant in Lobe 2 (RE-AA), which targeted site 3, slowed both t_{rise} and τ_{deact} to a greater extent (t_{rise} = 590 ± 50 μs and τ_{deact} = 4.9 ± 0.7 ms; $p < 0.05$, t test, n = 3; Figures 6B and 6C). For the RE-AA mutant, we also observed an apparent acceleration in recovery from desensitization (τ_{rec} = 13 ± 1 ms, n = 3; Figure S7) compared with WT GluA2, although again, poor expression made proper estimation of the recovery time constant difficult.

Off-Pathway Mutants Have Little Effect on Kinetics

Substantial charge swap mutations on the surface of the LBD could have non-specific effects on binding a charged ligand. In particular, we were concerned that triple mutations in general might slow binding, independent of the pathways we had identified. Therefore, as negative controls, we generated complementary, off-pathway mutations that our simulations predicted did not interact with glutamate during binding and unbinding. Two triple-alanine mutations were generated: K409A-K410A-E422A (denoted KKE-AAA) in Lobe 1 and R715A-K716A-D769A (denoted RKD-AAA) in Lobe 2. These off-pathway mutants had smaller effects on kinetics (t_{rise} = 100 ± 5 μs, τ_{deact} = 2.4 ± 0.1 ms for RKD; t_{rise} = 310 ± 10 μs, τ_{deact} = 1.1 ± 0.1 ms for KKE; Figures 6D–6F). Their kinetics conformed to previously published mutants that influence closed-cleft stability. Quite distinct from the metastable binding sites presented here, for RKD-AAA, slower deactivation was accompanied by faster activation (Table S3; indicative of an increase in affinity). For KKE-AAA, the results were inverted, with faster deactivation and slower activation (consistent with reduced glutamate affinity). More sophisticated tests of the pathways themselves in the context of the LBD surface will be the subject of future work.

For most of the mutants tested, the decay rate is slowed more profoundly than the opening rate. The decay time constant varies in an approximately linear fashion as a function of the activation time, and changes in the decay are roughly 8-fold larger than that of activation (Figure 7C). Simulated currents using previously published kinetic models of AMPA receptor activation (Robert et al., 2005) show that this relationship is precisely what is expected from slowing glutamate association and dissociation rates by equal amounts. Moreover, these calculations were performed with realistic concentration jump profiles (Lape et al., 2012), indicating the physically plausible solution exchange rate of about 300 μs would be expected to yield a very similar, roughly linear relationship between activation and deactivation

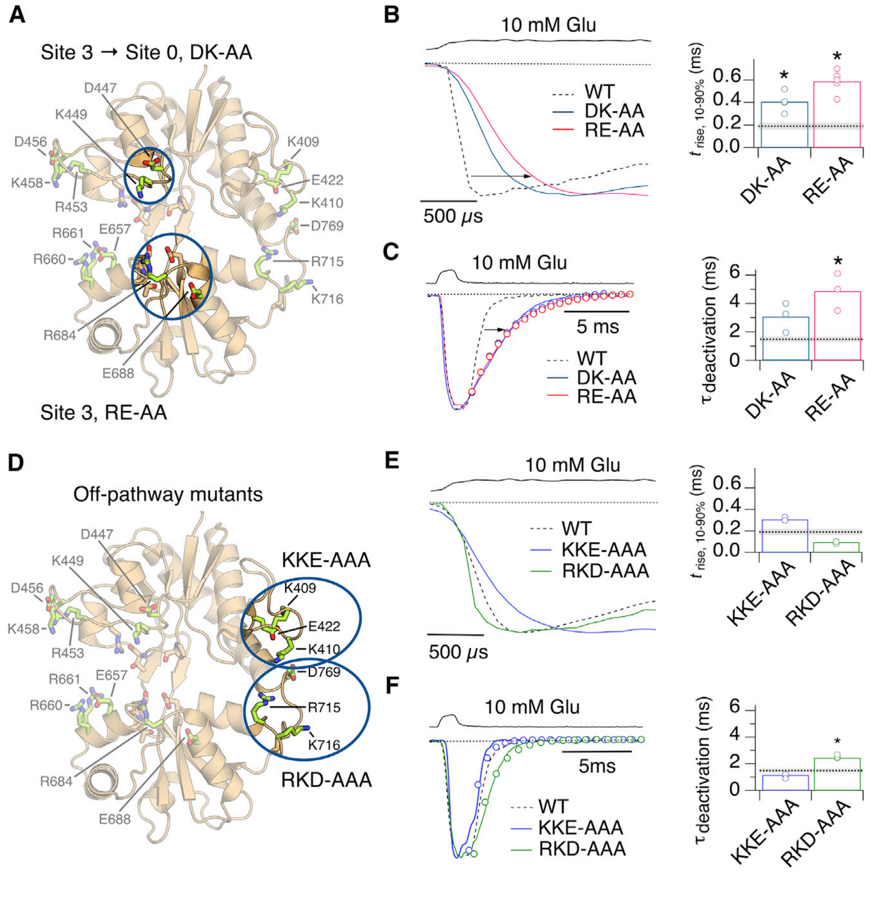

Figure 6. Activation and Deactivation of Receptors with Mutations in Pathway 3 and Off-Pathway Mutants

(A) The blue ovals indicate residues of the LBD that interact with the ligand. K449 participates in transferring the ligand from metastable site 3 to site 0 (Figure 2B). D447 and K449 were mutated to alanine (DK-AA). R684 participates in site 3, and two residues, R684 and E688, were separately mutated to alanine (RE-AA).

(B) The activation of receptors in response to a long pulse of 10 mM glutamate was slower than WT GluA2 (dashed line) for both DK-AA (blue trace) and RE-AA (red trace). The upper black trace shows solution exchange. Individual 10%–90% rise times are plotted in the right panel with the mean value for WT GluA2 indicated by a dashed line and standard error shaded in light gray. Asterisks indicate $p < 0.01$ versus WT GluA2, t test, n = 3–6.

(C) Deactivation of DK-AA and RE-AA mutants in response to a 1 ms pulse of 10 mM glutamate with color coding as in (B). Monoexponential fits are represented by open circles. Individual deactivation decay constants are plotted in the bar graph (right). The asterisk indicates $p < 0.05$ versus WT GluA2, t test, n = 3–6.

(D) Blue ovals indicate positions in the LBD of two triple-alanine mutants, located away from the binding pathways (Figure 2A). One set of off-pathway mutants is located in Lobe 1 (K409A-K410A-E422A, KKE-AAA) and one set in Lobe 2 (R715A-K716A-D769A, RKD-AAA) (Figure 4A).

(E) Left panel shows activation of receptors by long pulses of 10 mM glutamate. Responses for the KKE-AAA (blue trace) and RKD-AAA (green trace) mutants are overlaid with a typical WT GluA2 response as in (C), with individual rise times plotted in the right panel. Asterisk indicates $p < 0.05$.

(F) Left panel shows deactivation of off-pathway mutants following a 1 ms pulse of 10 mM glutamate. Color coding as in (E). Individual rise times are plotted in the right panel. Asterisk indicates $p < 0.01$.

See also Figure S7 and Table S3.

times (Figure 7). Notably, the off-pathway mutants KKE-AAA and RKD-AAA (Figures 6D–6F) lie away from the linear relation between activation and decay time (Figure 7C).

Taken together, these results strongly support the hypothesis that these metastable sites predicted *in silico* form preferential pathways to guide glutamate in and out of its binding site.

DISCUSSION

By combining long, unbiased molecular simulations with direct measurements of receptor kinetics, we show a key role for charged residues in facilitating fast neurotransmitter access to a deep binding site. These studies suggest that neurotransmitter binding is a directed process for which kinetics have been optimized (presumably by evolution) without altering overall ligand affinity. Previous work has shown that electrodiffusion of glutamate in the synaptic cleft speeds up neurotransmission (Sylantyev et al., 2008). Our experiments reveal a strikingly elaborate management of ligand transport by AMPA receptors, whereby flexible positive charges ensure that glutamate binding reactions are fast. The existence of these pathways is surprising, and the fact that they alter the kinetics of receptor activity indicates that the molecular mechanisms that determine the action of neurotransmitters at receptors are more complex than previously thought. R660 is conserved between AMPA and NMDA receptors; in kainate receptors, R660 and R661 are replaced by lysine residues (Figure S8). It is possible that these helix F interactions also coordinate ligand binding in kainate and NMDA receptors. Given that electrostatic interactions are also important for coordination in other neurotransmitter binding sites (McCammon, 2009), these principles of ligand funneling may be general.

STAR★METHODS

Detailed methods are provided in the online version of this paper and include the following:

- KEY RESOURCES TABLE
- CONTACT FOR REAGENT AND RESOURCE SHARING
- EXPERIMENTAL MODEL AND SUBJECT DETAILS
  - HEK293 Cell Cultures and Transfections
- METHOD DETAILS
  - Simulation System Preparation

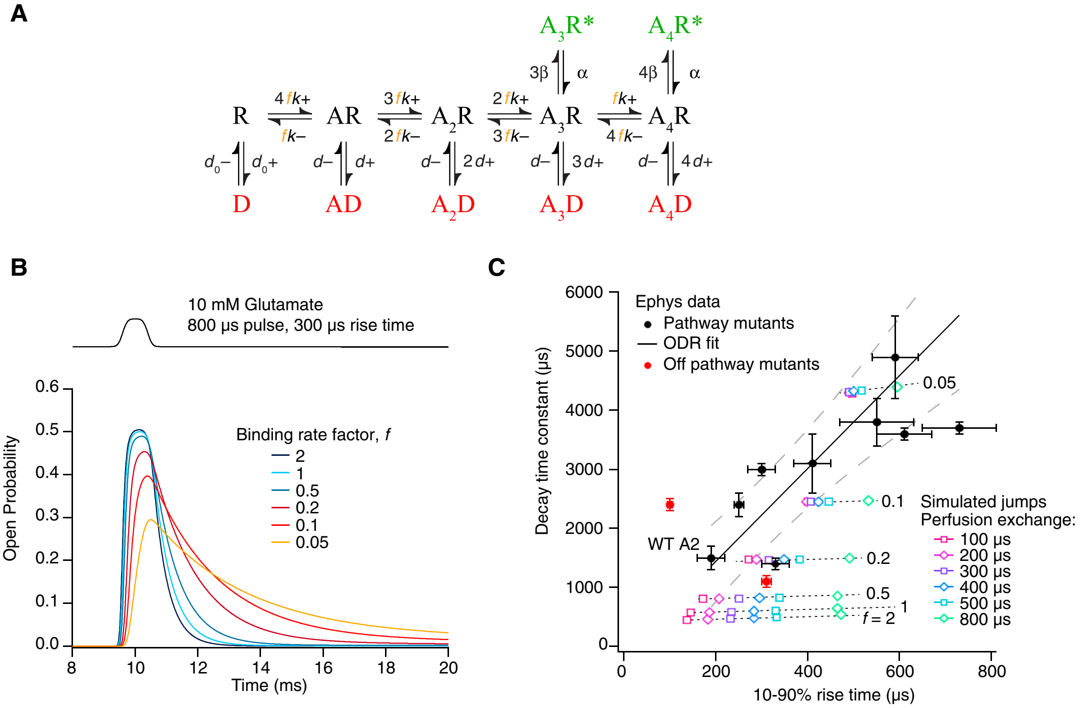

Figure 7. Kinetic Modeling of AMPA Receptor Currents Activated by Glutamate Recapitulates the Effect of Slowed Binding Reactions on Receptor Activation and Deactivation

(A) Kinetic model constructed according to principles outlined in previous studies of GluA2 kinetics (Robert et al., 2005). Four glutamate-binding sites, two open states (green, *), and five desensitized states (red) are included. Low conductance open states and connections between desensitized states were omitted for simplicity. For each simulation, the association and dissociation rates were multiplied by a common factor, *f*, to represent the effects of mutants to slow binding rates through disruption of glutamate binding pathways. The rate constants were as follows: beta = 5,000 s^{-1}, alpha = 3,000 s^{-1}, $k+ = 5 \times 10^6\ M^{-1}\ s^{-1}$, $k- = 10{,}000\ s^{-1}$, $d+ = 250\ s^{-1}$, $d- = 60\ s^{-1}$, $d_0+ = 1\ s^{-1}$, $d_0- = 9\ s^{-1}$. The conductance of the open state A4R* was set at twice that of A3R*.

(B) Example simulated currents (normalized to the maximum possible response) for a realistic concentration jump of 800 µs with rise time of 300 µs, using the model in (A). Six color-coded current profiles generated using the RCJ scripts (see STAR Methods) are shown, with the binding rate factor ranging from 2 to 0.05. Note that deactivation is more strongly affected than activation (because efficacy for channel opening is >1). Also, for fast binding reactions, the rise time is faster than the solution exchange.

(C) Kinetic measurements from GluA2 mutants and WT (black circles, WT GluA2 marked) were well described by a linear fit with slope of ~8 with intercept close to the origin. 95% confidence intervals for the line are shown as gray dashed curves. Uncertainties in both abscissa and ordinate were used for the fit (ODR = 2 in Igor 7). The reduced chi-square value for this fit is ~6, meaning that the linear description of the data is, at best, approximate. Off-pathway control mutants (red circles) lie away from this curve. Data from simulations with different rise times for the glutamate pulse are plotted as open symbols, with the relevant *f* value for each group of simulations indicated with a dotted line. The colors of these symbols relates to the simulated 10%–90% solution exchange time seen by receptors. All solution exchange rates predict the similar steep, approximately linear relations between activation time (10%–90%) and decay time constant. The simulated kinetics span a similar range to the electrophysiological recordings of pathway-disruption mutants. The best agreement between simulation and experiment comes from solution exchange times at an intact patch in the physically plausible range of 200–400 µs.

- ○ System Preparation for the ERR-AAA Simulations
- ○ Simulations with Increased Ligand Concentration
- ○ MD Simulations
- ○ Molecular Biology
- ○ Electrophysiology
- ● QUANTIFICATION AND STATISTICAL ANALYSIS
  - ○ Ligand Binding PMF
  - ○ Calculation of k_{on} from Molecular Simulations
  - ○ Functional Data Analysis
- ● DATA AND SOFTWARE AVAILABILITY

SUPPLEMENTAL INFORMATION

Supplemental Information includes eight figures, four tables, and four movies and can be found with this article online at https://doi.org/10.1016/j.neuron.2017.11.024.

AUTHOR CONTRIBUTIONS

A.Y. and A.Y.L. designed the molecular simulations and analyzed the data. A.Y. performed the molecular simulations. H.S. and A.J.R.P. designed the electrophysiology and analyzed the data, H.S. performed the electrophysiology, and A.J.R.P. performed the kinetic modeling. A.Y., H.S., A.J.R.P., and A.Y.L. wrote the paper.

ACKNOWLEDGMENTS

We thank Jelena Baranovic for comments on the manuscript. Anton computer time was provided by the Pittsburgh Supercomputing Center (PSC) through Grant R01GM116961 from the National Institutes of Health. The Anton machine at PSC was generously made available by D.E. Shaw Research. Resources provided by the Maryland Advanced Research Computing Center (MARCC) at Johns Hopkins University were used. This work was supported by the National Institutes of Health grant R01GM094495 (to A.Y.L.), the ERC grant

CellPress

"GluActive" (647895), and the DFG Cluster of Excellence "NeuroCure" (EXC-257) (to A.J.R.P.). A.J.R.P. is a Heisenberg Professor of the DFG (PL619-3.1). H.S. was the recipient of a fellowship from the Human Frontier Science Program (LT000079/2011-L).

Received: August 2, 2017
Revised: September 26, 2017
Accepted: November 15, 2017
Published: December 14, 2017

REFERENCES

Abele, R., Keinanen, K., and Madden, D.R. (2000). Agonist-induced isomerization in a glutamate receptor ligand-binding domain. A kinetic and mutagenetic analysis. J. Biol. Chem. *275*, 21355–21363.

Alberty, R.A., and Hammes, G.G. (1958). Application of the theory of diffusion-controlled reactions to enzyme kinetics. J. Phys. Chem. *62*, 154–159.

Arinaminpathy, Y., Sansom, M.S.P., and Biggin, P.C. (2002). Molecular dynamics simulations of the ligand-binding domain of the ionotropic glutamate receptor GluR2. Biophys. J. *82*, 676–683.

Armstrong, N., and Gouaux, E. (2000). Mechanisms for activation and antagonism of an AMPA-sensitive glutamate receptor: crystal structures of the GluR2 ligand binding core. Neuron *28*, 165–181.

Attwell, D., and Gibb, A. (2005). Neuroenergetics and the kinetic design of excitatory synapses. Nat. Rev. Neurosci. *6*, 841–849.

Baranovic, J., and Plested, A.J.R. (2016). How to build the fastest receptor on earth. Biol. Chem. *397*, 195–205.

Brooks, B.R., Brooks, C.L., 3rd, Mackerell, A.D., Jr., Nilsson, L., Petrella, R.J., Roux, B., Won, Y., Archontis, G., Bartels, C., Boresch, S., et al. (2009). CHARMM: the biomolecular simulation program. J. Comput. Chem. *30*, 1545–1614.

Cheng, Q., Du, M., Ramanoudjame, G., and Jayaraman, V. (2005). Evolution of glutamate interactions during binding to a glutamate receptor. Nat. Chem. Biol. *1*, 329–332.

Chou, K.C., and Zhou, G.P. (1982). Role of the protein outside active site on the diffusion-controlled reaction of enzymes. J. Am. Chem. Soc. *104*, 1409–1413.

Clements, J.D., Lester, R.A., Tong, G., Jahr, C.E., and Westbrook, G.L. (1992). The time course of glutamate in the synaptic cleft. Science *258*, 1498–1501.

Dai, J., and Zhou, H.-X. (2016). Semiclosed conformations of the ligand-binding domains of NMDA receptors during stationary gating. Biophys. J. *111*, 1418–1428.

Dror, R.O., Pan, A.C., Arlow, D.H., Borhani, D.W., Maragakis, P., Shan, Y., Xu, H., and Shaw, D.E. (2011). Pathway and mechanism of drug binding to G-protein-coupled receptors. Proc. Natl. Acad. Sci. USA *108*, 13118–13123.

Fiser, A., and Sali, A. (2003). ModLoop: automated modeling of loops in protein structures. Bioinformatics *19*, 2500–2501.

Jorgensen, W.L., Chandrasekhar, J., Madura, J.D., Impey, R.W., and Klein, M.L. (1983). Comparison of simple potential functions for simulating liquid water. J. Chem. Phys. *79*, 926.

Kräutler, V., van Gunsteren, W.F., and Hünenberger, P.H. (2001). A fast SHAKE algorithm to solve distance constraint equations for small molecules in molecular dynamics simulations. J. Comput. Chem. *22*, 501–508.

Krivov, G.G., Shapovalov, M.V., and Dunbrack, R.L., Jr. (2009). Improved prediction of protein side-chain conformations with SCWRL4. Proteins *77*, 778–795.

Lape, R., Plested, A.J.R., Moroni, M., Colquhoun, D., and Sivilotti, L.G. (2012). The α1K276E startle disease mutation reveals multiple intermediate states in the gating of glycine receptors. J. Neurosci. *32*, 1336–1352.

Lau, A.Y., and Roux, B. (2007). The free energy landscapes governing conformational changes in a glutamate receptor ligand-binding domain. Structure *15*, 1203–1214.

Lau, A.Y., and Roux, B. (2011). The hidden energetics of ligand binding and activation in a glutamate receptor. Nat. Struct. Mol. Biol. *18*, 283–287.

Lisman, J.E., Raghavachari, S., and Tsien, R.W. (2007). The sequence of events that underlie quantal transmission at central glutamatergic synapses. Nat. Rev. Neurosci. *8*, 597–609.

MacKerell, A.D., Bashford, D., Bellott, M., Dunbrack, R.L., Evanseck, J.D., Field, M.J., Fischer, S., Gao, J., Guo, H., Ha, S., et al. (1998). All-atom empirical potential for molecular modeling and dynamics studies of proteins. J. Phys. Chem. B *102*, 3586–3616.

Mackerell, A.D., Jr., Feig, M., and Brooks, C.L., 3rd (2004). Extending the treatment of backbone energetics in protein force fields: limitations of gas-phase quantum mechanics in reproducing protein conformational distributions in molecular dynamics simulations. J. Comput. Chem. *25*, 1400–1415.

Mamonova, T., Yonkunas, M.J., and Kurnikova, M.G. (2008). Energetics of the cleft closing transition and the role of electrostatic interactions in conformational rearrangements of the glutamate receptor ligand binding domain. Biochemistry *47*, 11077–11085.

Mayer, M.L. (2011). Emerging models of glutamate receptor ion channel structure and function. Structure *19*, 1370–1380.

Mayer, M.L. (2016). Structural biology of glutamate receptor ion channel complexes. Curr. Opin. Struct. Biol. *41*, 119–127.

McCammon, J.A. (2009). Darwinian biophysics: electrostatics and evolution in the kinetics of molecular binding. Proc. Natl. Acad. Sci. USA *106*, 7683–7684.

Mendieta, J., Gago, F., and Ramírez, G. (2005). Binding of 5′-GMP to the GluR2 AMPA receptor: insight from targeted molecular dynamics simulations. Biochemistry *44*, 14470–14476.

Okada, O., Odai, K., Sugimoto, T., and Ito, E. (2012). Molecular dynamics simulations for glutamate-binding and cleft-closing processes of the ligand-binding domain of GluR2. Biophys. Chem. *162*, 35–44.

Pan, A.C., Xu, H., Palpant, T., and Shaw, D.E. (2017). Quantitative characterization of the binding and unbinding of millimolar drug fragments with molecular dynamics simulations. J. Chem. Theory Comput. *13*, 3372–3377.

Phillips, J.C., Braun, R., Wang, W., Gumbart, J., Tajkhorshid, E., Villa, E., Chipot, C., Skeel, R.D., Kalé, L., and Schulten, K. (2005). Scalable molecular dynamics with NAMD. J. Comput. Chem. *26*, 1781–1802.

Postila, P.A., Swanson, G.T., and Pentikäinen, O.T. (2010). Exploring kainate receptor pharmacology using molecular dynamics simulations. Neuropharmacology *58*, 515–527.

Robert, A., and Howe, J.R. (2003). How AMPA receptor desensitization depends on receptor occupancy. J. Neurosci. *23*, 847–858.

Robert, A., Armstrong, N., Gouaux, J.E., and Howe, J.R. (2005). AMPA receptor binding cleft mutations that alter affinity, efficacy, and recovery from desensitization. J. Neurosci. *25*, 3752–3762.

Rosenmund, C., Stern-Bach, Y., and Stevens, C.F. (1998). The tetrameric structure of a glutamate receptor channel. Science *280*, 1596–1599.

Rudolph, S., Tsai, M.-C., von Gersdorff, H., and Wadiche, J.I. (2015). The ubiquitous nature of multivesicular release. Trends Neurosci. *38*, 428–438.

Sahai, M.A., and Biggin, P.C. (2011). Quantifying water-mediated protein-ligand interactions in a glutamate receptor: a DFT study. J. Phys. Chem. B *115*, 7085–7096.

Shan, Y., Klepeis, J.L., Eastwood, M.P., Dror, R.O., and Shaw, D.E. (2005). Gaussian split Ewald: a fast Ewald mesh method for molecular simulation. J. Chem. Phys. *122*, 54101.

Shaw, D.E., Dror, R.O., Salmon, J.K., Grossman, J.P., Mackenzie, K.M., Bank, J.A., Young, C., Deneroff, M.M., Batson, B., Bowers, K.J., et al. (2009). Millisecond-Scale Molecular Dynamics Simulations on Anton. In Proceedings of the ACM/IEEE Conference on Supercomputing (ACM), 1–11.

Shaw, D.E., Grossman, J.P., Bank, J.A., Batson, B., Butts, J.A., Chao, J.C., Deneroff, M.M., Dror, R.O., Even, A., Fenton, C.H., et al. (2014).

Anton 2: Raising the Bar for Performance and Programmability in a Special-Purpose Molecular Dynamics Supercomputer (IEEE), pp. 41–53.

Speranskiy, K., and Kurnikova, M. (2005). On the binding determinants of the glutamate agonist with the glutamate receptor ligand binding domain. Biochemistry *44*, 11508–11517.

Sun, Y., Olson, R., Horning, M., Armstrong, N., Mayer, M., and Gouaux, E. (2002). Mechanism of glutamate receptor desensitization. Nature *417*, 245–253.

Sylantyev, S., Savtchenko, L.P., Niu, Y.-P., Ivanov, A.I., Jensen, T.P., Kullmann, D.M., Xiao, M.-Y., and Rusakov, D.A. (2008). Electric fields due to synaptic currents sharpen excitatory transmission. Science *319*, 1845–1849.

Traynelis, S.F., Wollmuth, L.P., McBain, C.J., Menniti, F.S., Vance, K.M., Ogden, K.K., Hansen, K.B., Yuan, H., Myers, S.J., and Dingledine, R. (2010). Glutamate receptor ion channels: structure, regulation, and function. Pharmacol. Rev. *62*, 405–496.

Tuckerman, M., Berne, B.J., and Martyna, G.J. (1992). Reversible multiple time scale molecular dynamics. J. Chem. Phys. *97*, 1990.

Wade, R.C., Gabdoulline, R.R., Lüdemann, S.K., and Lounnas, V. (1998). Electrostatic steering and ionic tethering in enzyme-ligand binding: insights from simulations. Proc. Natl. Acad. Sci. USA *95*, 5942–5949.

Weston, M.C., Gertler, C., Mayer, M.L., and Rosenmund, C. (2006). Interdomain interactions in AMPA and kainate receptors regulate affinity for glutamate. J. Neurosci. *26*, 7650–7658.

Wolter, T., Steinbrecher, T., and Elstner, M. (2013). Computational study of synthetic agonist ligands of ionotropic glutamate receptors. PLoS ONE *8*, e58774.

Yao, Y., Belcher, J., Berger, A.J., Mayer, M.L., and Lau, A.Y. (2013). Conformational analysis of NMDA receptor GluN1, GluN2, and GluN3 ligand-binding domains reveals subtype-specific characteristics. Structure *21*, 1788–1799.

Yu, A., Alberstein, R., Thomas, A., Zimmet, A., Grey, R., Mayer, M.L., and Lau, A.Y. (2016). Molecular lock regulates binding of glycine to a primitive NMDA receptor. Proc. Natl. Acad. Sci. USA *113*, E6786–E6795.

Zhu, F., and Hummer, G. (2012). Convergence and error estimation in free energy calculations using the weighted histogram analysis method. J. Comput. Chem. *33*, 453–465.

CellPress

STAR★METHODS

KEY RESOURCES TABLE

REAGENT or RESOURCE	SOURCE	IDENTIFIER
Experimental Models: Cell Lines		
Human: HEK293 cell line, Female	DSMZ (German Collection of Cell Cultures and Microorganisms	300192/p777_HEK293
Oligonucleotides		
Primer: R453D Forward: TATGGGGCCGATGATGCCGAC	This paper	N/A
Primer: R453D Reverse: GTCGGCATCATCGGCCCCATA	This paper	N/A
Primer: K458D Forward: GATGCCGACACCGATATTTGG	This paper	N/A
Primer: K458D Reverse: CCAAATATCGGTGTCGGCATC	This paper	N/A
Primer: RDK-AAA Forward: TATGGGGCCGCTGATGCCGCTACCGCTATTTGG	This paper	N/A
Primer: RDK-AAA Reverse: CCAAATAGCGGTAGCGGCATCAGCGGCCCCATA	This paper	N/A
Primer: R660E Forward: GAGTTTTTCGGAAGATCTAAAATCGCAGTG	This paper	N/A
Primer: R660E Reverse: CACTGCGATTTTAGATCTTCCGAAAAACTC	This paper	N/A
Primer: R661E Forward: GAGTTTTTCAGGGGATCTAAAATCGCAGTG	This paper	N/A
Primer: R661E Reverse: CACTGCGATTTTAGATCCCCTGAAAAACTC	This paper	N/A
Primer: ERR-AAA Forward: GGCTCCACTAAAGCTTTTTTCGCTGCTTCTAAAATCGC	This paper	N/A
Primer: ERR-AAA Reverse: GCGATTTTAGAAGCAGCGAAAAAAGCTTTAGTGGAGCC	This paper	N/A
Primer: DK-AA Forward: ATTGTTGGGGCTGGCGCTTATGGGGCC	This paper	N/A
Primer: DK-AA Reverse: GGCCCCATAAGCGCCAGCCCCAACAAT	This paper	N/A
Primer: RE-AA Forward: GTGTTTGTGGCTACTACCGCAGCTGGAGTAGCC	This paper	N/A
Primer: RE-AA Reverse: GGCTACTCCAGCTGCGGTAGTAGCCACAAACAC	This paper	N/A
Primer: K409A, K410A Forward (for KKE-AAA): GTTATGATGGCTGCTAATCATGAA	This paper	N/A
Primer: K409A, K410A Reverse (for KKE-AAA): TTCATGATTAGCAGCCATCATAAC	This paper	N/A
Primer: E422A Forward (for KKE-AAA): GAGCGTTACGCTGGCTACTGT	This paper	N/A
Primer: E422A Reverse (for KKE-AAA): ACAGTAGCCAGCGTAACGCTC	This paper	N/A
Primer: R715A, K716A Forward (for RKD-AAA): ATCGAGCAGGCTGCTCCTTGTGAC	This paper	N/A
Primer: R715A, K716A Reverse (for RKD-AAA): GTCACAAGGAGCAGCCTGCTCGAT	This paper	N/A

(Continued on next page)

Continued		
REAGENT or RESOURCE	SOURCE	IDENTIFIER
Primer: D769A Forward (for RKD-AAA): TGGTGGTACGCTAAAGGTGAATGT	This paper	N/A
Primer: D769A Reverse (for RKD-AAA): ACATTCACCTTTAGCGTACCACCA	This paper	N/A
Recombinant DNA		
GluA2_IRES_eGFP pRK5 vector	Sun et al., 2002	Mark Mayer's lab
Software and Algorithms		
CHARMM	Brooks et al., 2009	https://www.charmm.org/
NAMD 2.9	UIUC	http://www.ks.uiuc.edu/Research/namd/
VMD 1.9.1	UIUC	http://www.ks.uiuc.edu/Research/vmd/
Pymol	Schrödinger, LLC	https://www.pymol.org
Realistic Concentration Jumps (RCJ) scripts	Lape et al., 2012; this paper	https://github.com/aplested/aligator
IGOR Pro	IGOR Pro	RRID: SCR_000325; http://www.wavemetrics.com/products/igorpro/igorpro.htm
Axograph	Axograph	RRID: SCR_014284; https://www.axograph.com/
Adobe Illustrator	Adobe Systems	RRID: SCR_010279; http://www.adobe.com/products/illustrator.html
Adobe Photoshop	Adobe Systems	RRID: SCR_014199; https://www.adobe.com/products/photoshop.html

CONTACT FOR REAGENT AND RESOURCE SHARING

Further information and requests for resources and reagents should be directed to and will be fulfilled by the Lead Contact, Albert Lau (alau@jhmi.edu).

EXPERIMENTAL MODEL AND SUBJECT DETAILS

HEK293 Cell Cultures and Transfections

HEK293 cells, female, were maintained in DMEM (GIBCO) supplemented with 10% fetal bovine serum (FBS) and 1% penicillin-streptomycin. For transfection experiments, cells were seeded in 2 mL culture dishes and, 48 hr later, transfected with Calcium phosphate (Sigma) and 2 μg total plasmid DNA.

METHOD DETAILS

Simulation System Preparation

The initial atomic models for both the monomer and dimer systems were constructed from the crystal structure of the GluA2 ligand-binding core (S1S2) in complex with glutamate (PDB: 1FTJ). Missing amino acid residues were built using the Modloop server (Fiser and Sali, 2003), and missing side chains were built using SCWRL4 (Krivov et al., 2009). Crystallographic waters in the ligand-binding cleft were included. The monomer system, which contained a total of 47,227 atoms, was solvated with 14,369 water molecules and neutralized by adding Na^+ and Cl^- ions to the bulk solution until the salt concentration was 150 mM NaCl. Periodic boundary conditions were imposed on an orthorhombic cell with approximate dimensions 88 Å × 68 Å × 78 Å. The dimer system, which contained a total of 56,217 atoms, was solvated with 15,951 water molecules and neutralized to maintain 150 mM NaCl. Periodic boundary conditions were imposed on an orthorhombic unit cell of approximate dimensions 96 Å × 78 Å × 78 Å. The system was energy minimized and equilibrated using constant pressure and temperature (NPT) conditions at 1 atm and 300 K with a timestep of 2 fs. The all-atom potential energy function PARAM27 for proteins (MacKerell et al., 1998, 2004) and the TIP3P potential energy function for water (Jorgensen et al., 1983) were used. Electrostatic interactions were computed using the particle mesh Ewald (PME) algorithm and

short-range, non-bonded interactions were truncated to 12 Å. The initial protein configuration of the system was relaxed with Langevin dynamics in the presence of harmonic restraints at constant volume for 30 ps before the barostat was switched on at 1 atm for a further 60 ps of simulation in NPT conditions. The cell dimensions were allowed to vary for 2 ns in NPT conditions before reaching the final box size. A 4 ns pre-production run in constant volume and temperature (NVT) conditions was carried out from which five, ligand-bound, starting coordinates for the monomer, and one, ligand-bound, starting coordinates for the dimer were selected for long-timescale simulation. The pre-production run was performed using NAMD 2.9 (Phillips et al., 2005), while minimization and equilibration procedures were performed using CHARMM (Brooks et al., 2009).

System Preparation for the ERR-AAA Simulations

Starting coordinates were selected from trajectory T_{mon2}, which involved the monomer system containing 10 ligands. Residues E657, R660, and R661 were mutated to alanine in CHARMM by deleting the side chain atoms and replacing with methyl groups. The salt concentration was adjusted to maintain 150 mM NaCl. The mutant system containing 47,080 atoms was energy minimized and briefly equilibrated in a 2 ns pre-production run in constant NVT conditions.

Simulations with Increased Ligand Concentration

Glutamate ligand molecules were initially placed at random positions and orientations in bulk solvent, similar to the approach of Dror et al. (2011). For the LBD monomer, 10 ligands were added at arbitrary positions in bulk solvent, each greater than 20 Å from the binding pocket, to a previously prepared system containing an LBD in an open conformation, derived from the apo crystal structure (PDB: 1FTO). Salt concentration was adjusted for ligand charge to maintain 150 mM NaCl. A 2 ns pre-production run was carried out in constant NVT conditions. For the LBD dimer, 20 ligands were added at arbitrary positions in bulk solvent greater than 20 Å from the binding pocket to a previously prepared system containing open conformations of the LBDs (PDB: 1FTO). Salt concentration was adjusted for ligand charge to maintain 150 mM NaCl. A 2 ns pre-production run was carried out in constant NVT conditions.

MD Simulations

All production runs used the NPT ensemble at 1 atm and 300K. Bond lengths for hydrogen atoms were constrained using the M-SHAKE algorithm (Kräutler et al., 2001). An r-RESPA integrator (Tuckerman et al., 1992) was used with a timestep of 2 fs; long-range electrostatics were computed every 6 fs. Long-range electrostatics interactions were calculated using the k-space Gaussian split Ewald method (Shan et al., 2005) with a 64 Å × 64 Å × 64 Å grid, $\sigma = 2.02$ Å, $\sigma_s = 1.29$ Å. Short-range interactions including van der Waals and short-range electrostatics were truncated at 9 Å. To prevent overall rotational and translational motion of the protein, positional harmonic restraints were applied on the backbone atoms of residues 426–428 (residues 37–39 in 1FTJ), residues 474–476 (residues 85–87 in 1FTJ), and residues 490–492 (residues 101–103 in 1FTJ) with a force constant of 0.3 kcal mol^{-1} $Å^{-2}$. All productions simulations for the WT protein were carried out on the special purpose Anton machine at the Pittsburgh Supercomputing Center (PSC) (Shaw et al., 2009). Production simulations for the ERR-AAA mutant protein were carried out on the Anton2 machine at the PSC (Shaw et al., 2014). Simulations on Anton2 were carried out as they were on Anton, except using a temperature of 310K, which is the default for Anton2. A total of 21 trajectories were generated for an aggregate simulation time of 49.1 μs: 11.8 μs involved WT dimers, 36.1 μs involved WT monomers, and 1.2 μs involved the ERR-AAA monomer.

Molecular Biology

The mutants for functional studies were generated by overlap PCR on the GluA2flip template (GI: 8393475) in the pRK5 vector. The cDNA encoded a Q at the Q/R editing site. For each mutant, the entire amplified cassette was confirmed by double-stranded DNA sequencing. Numbering refers to the mature polypeptide chain.

Electrophysiology

WT and mutant glutamate receptors were overexpressed in HEK293 cells using calcium phosphate transfection. The external solution contained: 150 mM NaCl, 0.1 mM $MgCl_2$, 0.1 mM $CaCl_2$, and 5 mM HEPES, titrated to pH 7.3 with NaOH, to which we added drugs as required. Drugs were obtained from Ascent Scientific and Sigma. The pipette solution contained: 115 mM NaCl, 10 mM NaF, 0.5 mM $CaCl_2$, 1 mM $MgCl_2$, 5 mM Na_4BAPTA, 5 mM HEPES and 10 mM Na_2ATP (pH 7.3). The sampling rate was 10 kHz (100 μs time step) and during acquisition the data were filtered at 5 kHz (10%–90% rise time 66 μs). We were restricted to a 5 kHz filter because of the low expression (and hence poor signal to noise) for some of the mutants. We applied ligands to outside out patches via a piezo-driven fast perfusion system. Typical 10%–90% solution exchange times were faster than 300 μs, as measured from junction potentials at the open tip of the patch pipette. Simulations using realistic concentration jumps were done as described (Lape et al., 2012) using a suite of PYTHON scripts (https://github.com/aplested/aligator). The effective decay constant was back-calculated from the 90%–10% decay time assuming a single exponential decay to facilitate comparison. These simulations showed that a solution exchange of 300 μs reproduces all the observed features of glutamate-activated AMPA receptor currents, including the approximately 200 μs rise time of wild-type channels (Figure 7).

QUANTIFICATION AND STATISTICAL ANALYSIS

Ligand Binding PMF

The trajectories containing the monomeric LBD with 10 ligands ($T_{mon2,3}$ and $T_{mut1\text{-}4}$) were sampled at 0.12 ns intervals. The monomeric LBD system was chosen to avoid conformational change between the LBD subunits affecting the density calculations. Systems with 10 ligands were chosen for increased ligand sampling. Cartesian coordinates for the ligand's non-hydrogen atoms, $\vec{r}$, were measured and used as three-dimensional order parameters to describe the states along the binding pathway. Each frame in the trajectory was aligned with respect to the backbone atoms of the LBD. The density of atomic positions, $\rho(\vec{r})$, was computed using a hard sphere van der Waals approximation onto a discretized grid with a spacing of 0.5 Å × 0.5 Å × 0.5 Å and subsequently weighted to produce the free energy maps using the standard Boltzmann re-weighting scheme, i.e., $\mathcal{W}(\vec{r}) = -k_B T\, ln[\rho(\vec{r})]$. Contour values for the PMF are indicated in the legends of Figures 2 and S2. The statistical uncertainty in the PMF was determined using the approach of block averaging (Zhu and Hummer, 2012) (Figure S2). The trajectory was subdivided into 10 blocks, and a PMF was calculated for each block. The standard deviation in the 10 PMFs was calculated. Using 5–15 blocks all gave qualitatively similar results.

Calculation of k_{on} from Molecular Simulations

$$k_{on} = \frac{N_b}{\sum_i \frac{t_i [L_i]}{s_i}}$$

where N_b is the total number of binding events, t_i is the time the ligand spends in bulk solvent, $[L_i]$ is the free ligand concentration, s_i is the number of protein subunits, and i is summed over all simulation systems. k_{on} calculated irrespective of the bound pose of the ligand is $1.4 \times 10^7\ M^{-1}\ s^{-1}$; k_{on} calculated for only the crystallographic pose is $0.5 \times 10^7\ M^{-1}\ s^{-1}$. These values suggest glutamate binding is not diffusion controlled since they are 100 times slower than the association rates of typical diffusion-limited binding processes (e.g., $k_{on} \sim 10^9 - 10^{10}\ M^{-1}\ s^{-1}$) (Alberty and Hammes, 1958; Chou and Zhou, 1982). All quantities used in the calculation are provided in Table S2. We did not obtain enough events to accurately calculate a dissociation constant (Pan et al., 2017). Electrostatic steering may play a role in determining the overall association rates of the ligand to the binding site (Wade et al., 1998).

Functional Data Analysis

To measure deactivation and desensitization decay constants, we fitted currents with a single exponential function. The rise times were interpolated from the 10%–90% crossing times of a sigmoid function fitted from the baseline to the peak current. This procedure gave an estimate for the wild-type GluA2 receptor rise time very similar to previously published work (Robert et al., 2005); almost all the mutants we report here had slower rise times. We measured concentration-response curves for WT and the mutant receptor EKK-AAA. We obtained the EC_{50} and maximum extent of activation relative to glutamate from fits to the Hill equation

$$\frac{I}{I_{max}} = \frac{[A]^n}{[A]^n + [EC_{50}]^n},$$

where n is the Hill coefficient, I_{max} is the maximum response, and [A] is the agonist concentration.

To measure recovery from desensitization, we used a two-pulse protocol with a variable interpulse interval. Recovery data were fitted by a Hodgkin-Huxley-type function (Robert and Howe, 2003)

$$N = N_0 + (1 - N_0)[1 - \exp(-k_{rec} t)]^n,$$

where N is the active fraction of receptors at time t following the first pulse, N_0 is the active fraction at the end of the conditioning pulse, and k_{rec} is the rate of recovery.

For all datasets, two-tailed unpaired Student's t tests were used to assess statistical significance between means, where a p value < 0.05 was considered significant. The number of experiments and statistical information are stated in the corresponding figure legends.

DATA AND SOFTWARE AVAILABILITY

Realistic Concentration Jumps (RCJ) scripts are available at https://github.com/aplested/aligator. Data are available upon request from the Lead Contact.

Neuron, Volume *97*

Supplemental Information

Neurotransmitter Funneling Optimizes Glutamate Receptor Kinetics

Alvin Yu, Héctor Salazar, Andrew J.R. Plested, and Albert Y. Lau

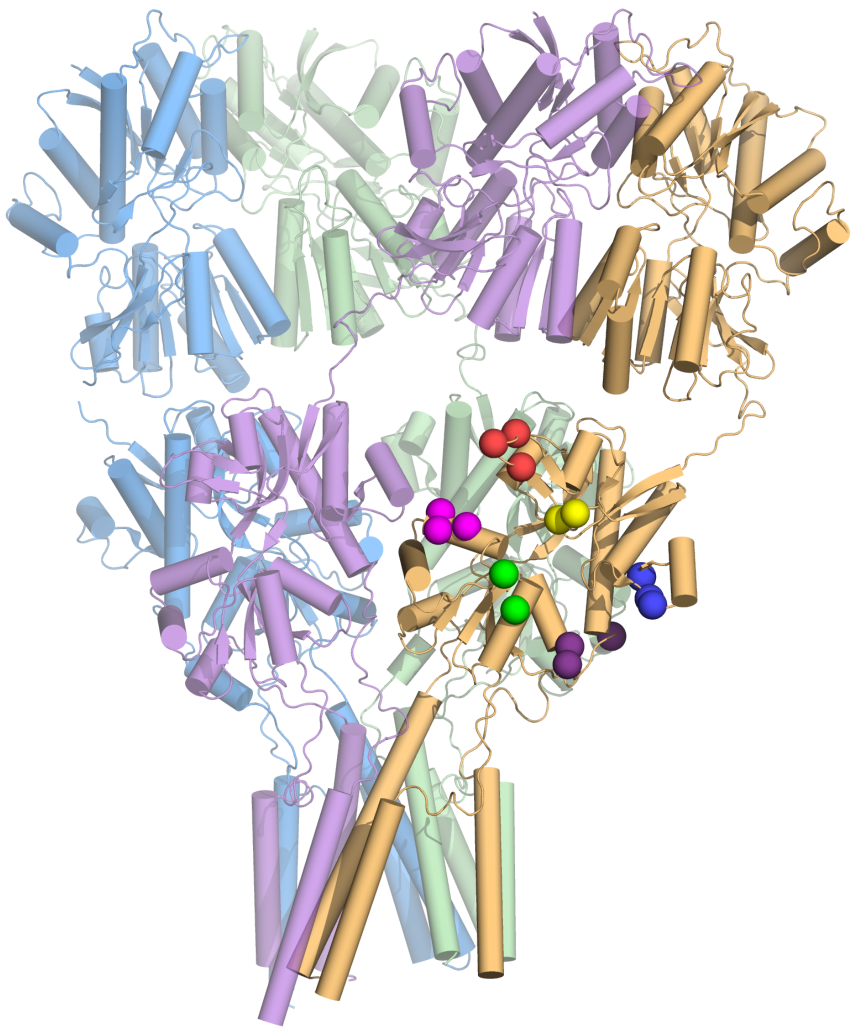

Figure S1. Sites of mutation in a tetrameric GluA2 receptor. Related to Figure 4.

Each sphere corresponds to a site of mutation shown in Figure 4a in a tetrameric receptor. R453, D456, and K458 are in red; E657, R660, and R661 are in magenta; D447 and K449 are in yellow; R684 and E688 are in green; K409, K410, and E422 are in blue; R715, K716, and D769 are in violet. The mutations occur in all subunits of the receptor, but they are shown in only one subunit for clarity. The structure shown is PDB ID 4U2P (Dürr et al., 2014).

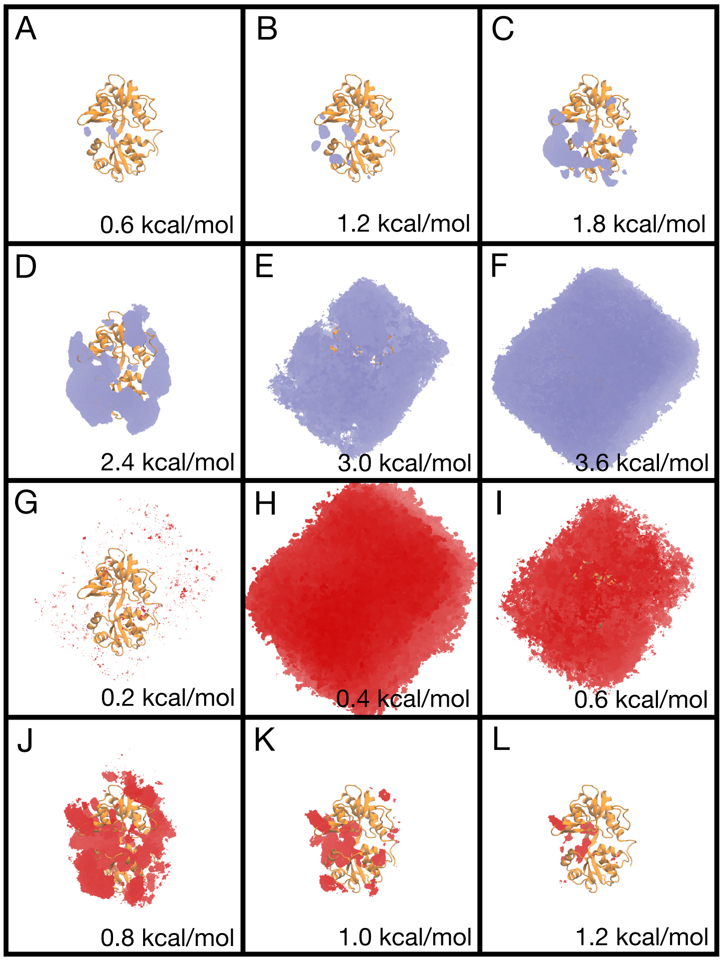

Figure S2. Error analysis of the ligand density PMF. Related to Figure 2. (a–f) The 3D PMF (blue) is shown at contour levels ranging from 0.6 kcal/mol to 3.6 kcal/mol in increments of 0.6 kcal/mol. **(g–l)** The statistical uncertainty in the 3D PMF determined using the approach of block averaging. 10 blocks were used. Contours of the 3D standard deviation (red) are shown from 0.2 kcal/mol to 1.2 kcal/mol in increments of 0.2 kcal/mol. Protein-ligand interactions (a–d) have uncertainties ranging from approximately ±0.8 to ±1.2 kcal/mol (g–l), whereas sites in bulk solvent (e, f) have lower uncertainties of approximately ±0.2 to ±0.6 kcal/mol (j-l).

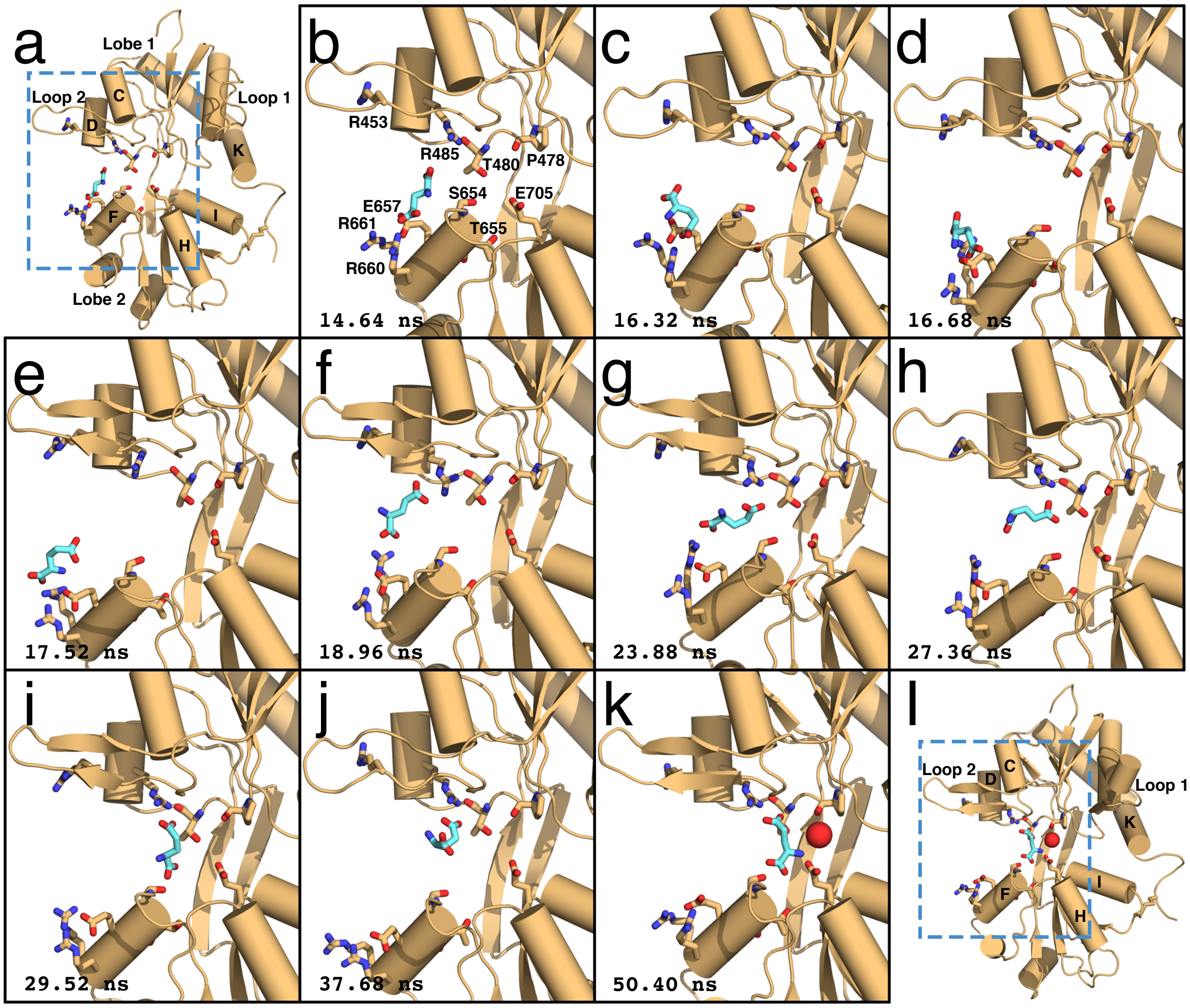

Figure S3. Glutamate binding in the inverted conformation. Related to Figure 3 and Movie S2. Time points (lower left of each panel) are relative to the start of Movie S2. **(a)** Prior to ligand binding, the LBD is closed; (ξ_1, ξ_2) = (11.0, 11.7 Å). The ligand's γ-carboxylate contacts R660. **(b)** Close-up view of **a**. **(c–d)** The ligand moves away from R485 as it is passed from R660 to R661. Interactions between the ligand and helix F residues switch from the γ-carboxylate at R660 to the α-carboxylate at R661. **(e)** The LBD opens, (ξ_1, ξ_2) = (15.0, 11.2 Å), to allow ligand entry into the binding pocket. **(f–g)**

The ligand metastably bridges Lobes 1 and 2 as its γ-carboxylate contacts R485, which has transiently rotated out of the binding pocket to form the interaction. **(h)** Interactions with helix F residues are broken, and the ligand moves into the binding pocket, tethered to R485. **(i)** Glutamate adopts the inverted bound conformation. **(j–k)** The ligand's amide contacts E705. A hydrogen bonding network is formed involving P478, a water molecule, and the ligand's amide, stabilizing the inverted conformation. **(l)** Expanded view of **k**. The LBD remains slightly open around the inverted conformation; (ξ_1, ξ_2) = (13.2, 11.1 Å). This trajectory corresponds to T_{mon1} in Table S1.

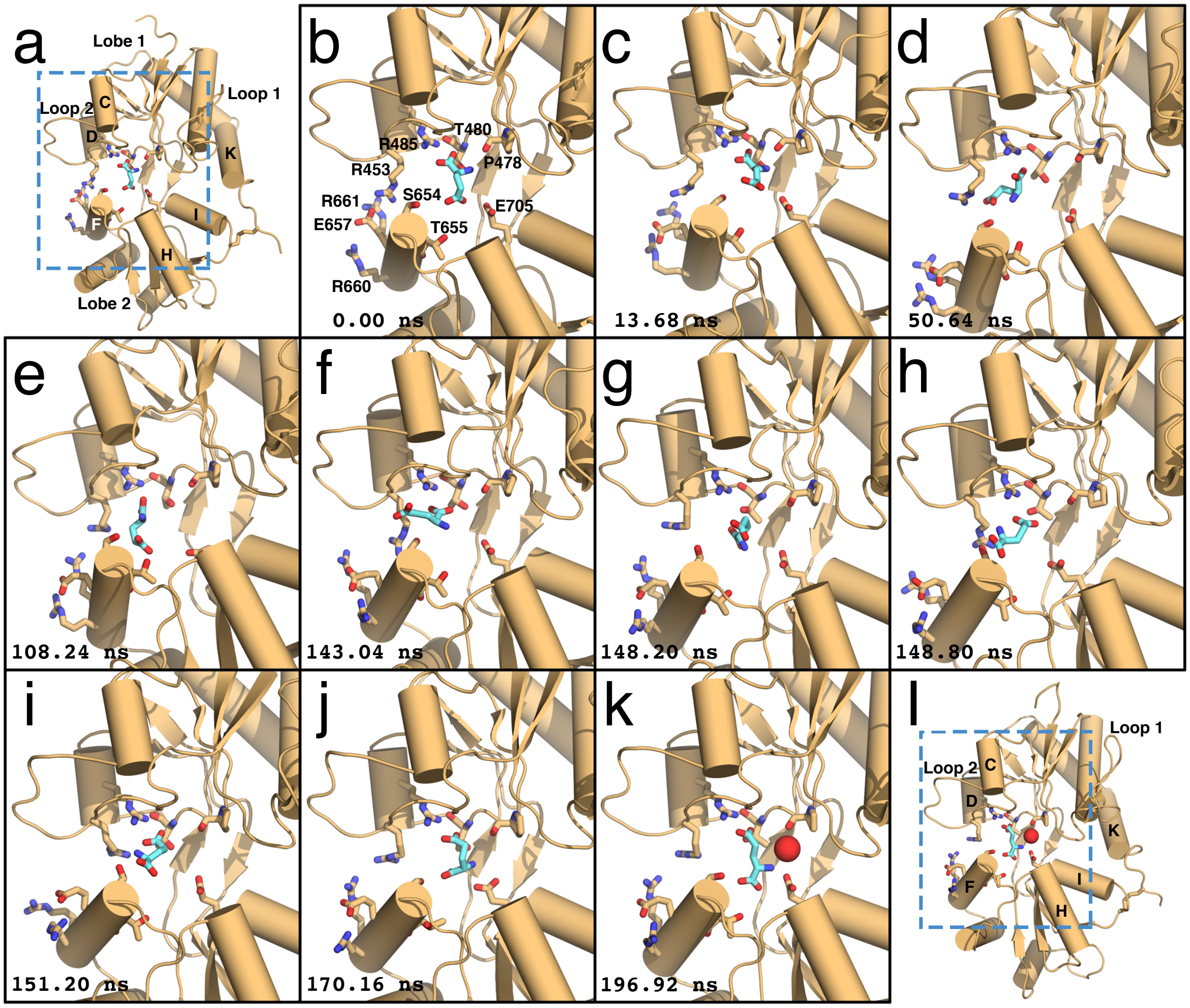

Figure S4. Interconversion between bound ligand conformations. Related to Figure 3 and Movie S3. Time points (lower left of each panel) are relative to the start of Movie S3. **(a)** Initially, the ligand binds in the crystallographic conformation. **(b)** Close-up view of **a**; the ligand's α-carboxylate contacts R485, whereas the amide is coordinated by P478 and E705. **(c–d)** The γ-carboxylate swings out of the binding pocket to contact R453. Interactions between the ligand's amide with P478 and E705 are broken. **(e–f)** The γ-carboxylate rotates freely in the binding cleft while the α-carboxylate remains

tethered to R485. **(g)** Contacts between the α-carboxylate and R485 are severed as the ligand repositions. **(h)** The γ-carboxylate of the ligand contacts R485. **(i)** The ligand swings into the binding pocket. **(j)** Glutamate adopts the inverted bound conformation. **(k)** The ligand's amide contacts E705, and P478 forms a hydrogen bond with a water molecule that stabilizes the inverted conformation. The ligand's α-carboxylate interacts with S654 in Lobe 2. **(l)** Expanded view of **k**. This trajectory corresponds to T_{dim2} in Table S1.

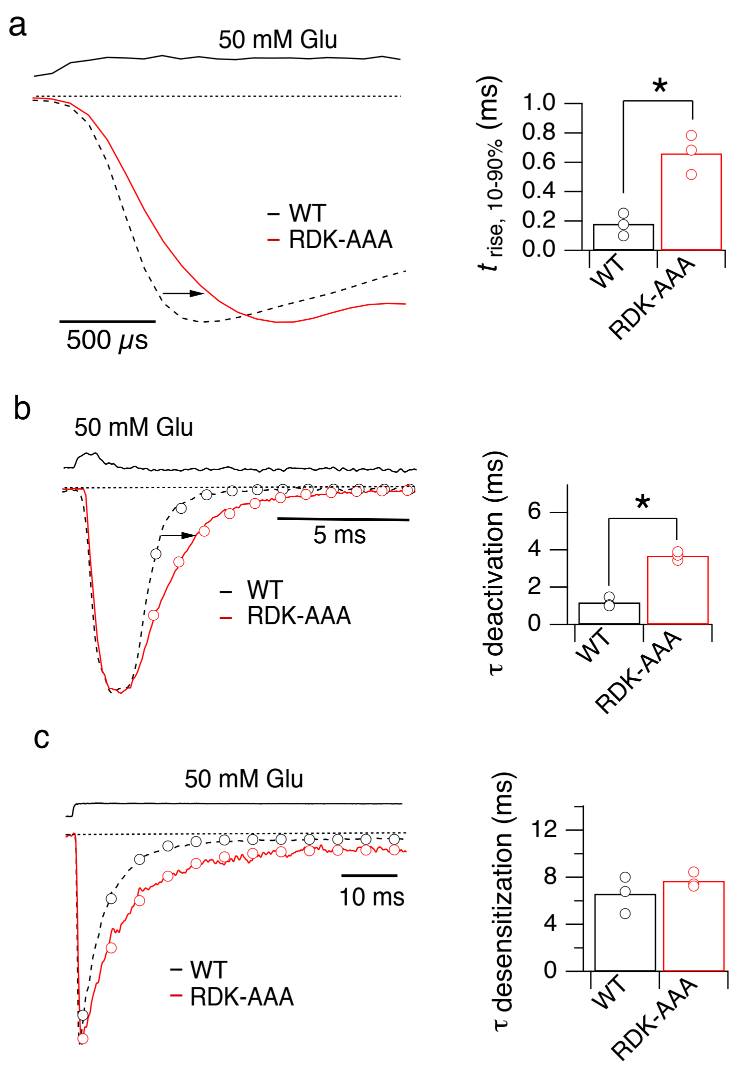

Figure S5. Slower activation and deactivation are retained in high glutamate. Related to Figure 4. (a) Normalized current responses corresponding to activation by a long pulse of 50 mM glutamate are shown for the RDK-AAA mutant (red) with WT GluA2 (dashed black trace). The upper trace shows the application of glutamate. Rise times (10-90%) from individual patches are shown in the bar graph (right panel). (p = 0.01 vs. WT; t-test, n = 3). **(b)** Monoexponential decays for WT GluA2 (dashed black line) and RDK-AAA (red line) were fitted for the deactivation in response to a 1 ms pulse of 50 mM glutamate (open circles). The upper trace shows the open tip response.

Deactivation time constants from individual patches are plotted in the bar graph (right panel; $p = 0.0003$; *t*-test, $n = 3$). **(c)** Desensitization time constants were not altered by the RDK-AAA mutant. Monoexponential fits as in **b**. Desensitization time constants from individual patches plotted in the bar graph (right panel) revealed no difference between WT and RDK-AAA ($p = 0.3$; *t*-test, $n = 3$).

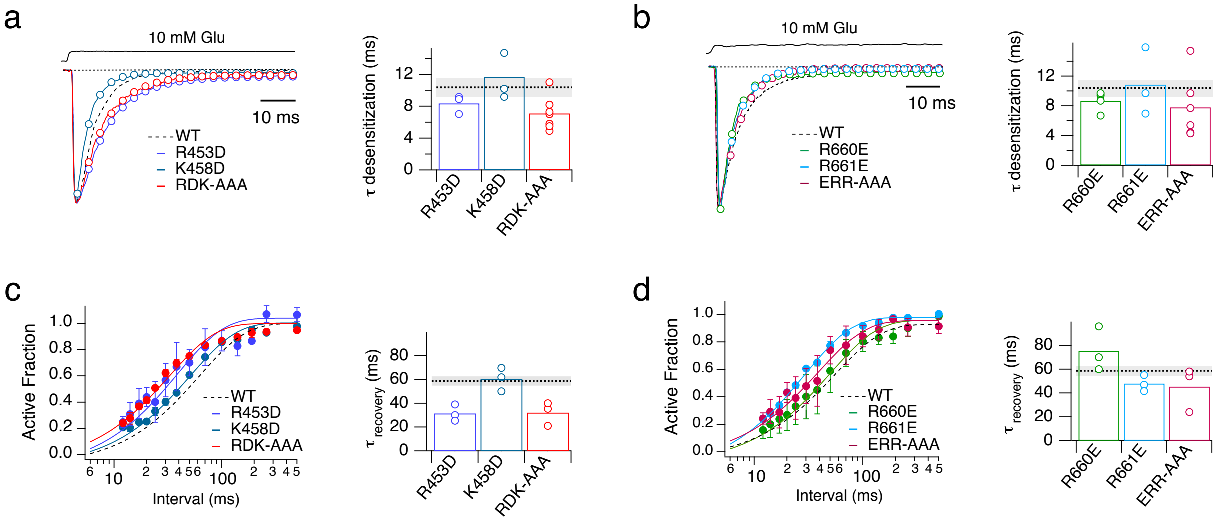

Figure S6. Desensitization and recovery from desensitization of receptors mutated at metastable sites 1 and 2. Related to Figures 4 and 5. (a) Monoexponential fits to desensitization decays of R453D, K458D and the R453D, D456A, K458A (RDK-AAA) mutants in response to 10 mM glutamate were similar to WT ($p > 0.03$; *t*-test, $n = 3 – 7$). A typical WT GluA2 response is shown as a dashed line. Decay constants for individual patches are plotted in the right panel, with the average value for WT GluA2 indicated by a black dashed line, with standard error shaded in light grey. **(b)** As for **a**, but for the R660E, R661E and E657A, R660A, R661A triple mutants. Fits were similar to that of WT ($p > 0.2$; *t*-test, $n = 3$). **(c)** Left panels show fits to pooled responses ("Active Fraction") at increasing intervals after a long pulse of 10 mM glutamate for WT and the different mutants. The R453D and RDK-AAA mutants recovered from desensitization about twice as fast as WT ($p < 0.02$; *t*-test, $n = 3$). Recovery time constants for individual patches, with the WT mean value and standard error indicated as in **a–b**. **(d)** Recovery data for the R660E, R661E and ERR-AAA

mutants presented as in **c**. Recovery time constants for these mutants were not significantly different from that of WT ($p > 0.1$; *t*-test, $n = 3$).

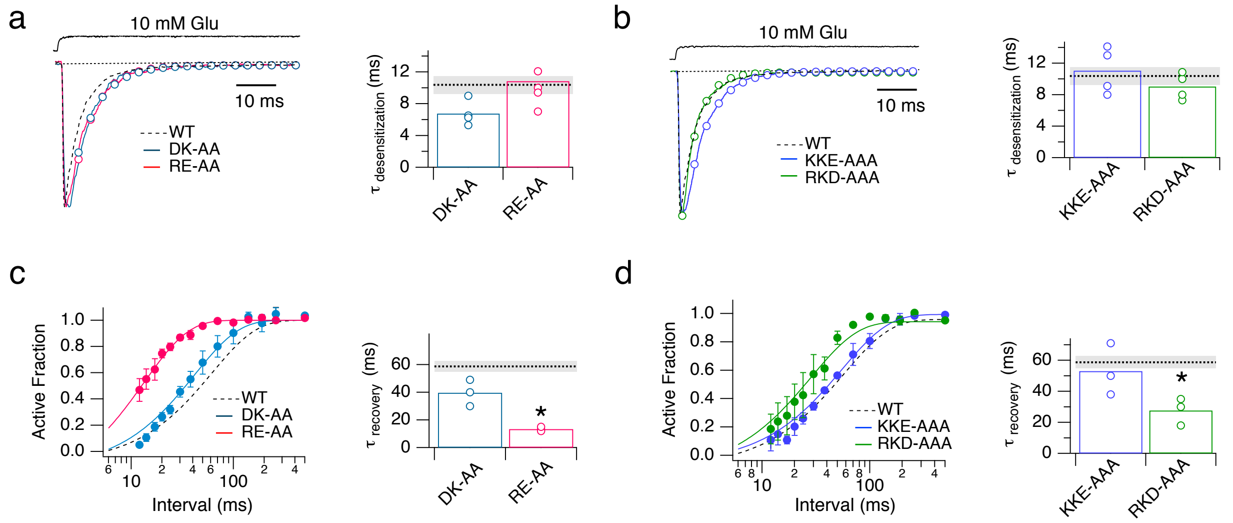

Figure S7. Desensitization and recovery from desensitization of receptors with mutations in Pathway 3 and in off-pathway regions. Related to Figure 6. (a) Monoexponential fits to desensitization decays of DK-AA (D447A K449A) and RE-AA (R684A, E688A) mutants in response to 10 mM glutamate were similar to WT ($p = 0.03$ for DK-AA, $p = 0.8$ for RE-AA; *t*-test, $n = 3–7$). Fits are represented by open circles. A typical WT GluA2 response is overlaid (black dashed line). Decay constants for individual patches are plotted in the right panel, with the average value for WT GluA2 indicated by a black dotted line, with its standard error shaded in light grey. **(b)** As for **a**, but for the KKE-AAA and RKD-AAA control mutants ($P = 0.7$ and 0.4 vs. WT, respectively; *t*-test). **(c)** Left panels show fits to pooled responses (“Active Fraction”) at increasing intervals after a long pulse of 10 mM glutamate for WT and the DK-AA and RE-AA mutants. The RE-AA mutant gave very small currents recovered from desensitization about four times as fast as WT ($p = 0.008$; *t*-test, $n = 3–5$). Recovery time constants for individual patches, with the WT mean value and standard error

indicated as in **a–b**. **(d)** Recovery data for the KKE-AAA and RKD-AAA mutants (P = 0.6 and 0.01 vs. WT, respectively; *t*-test), presented as in **c**.

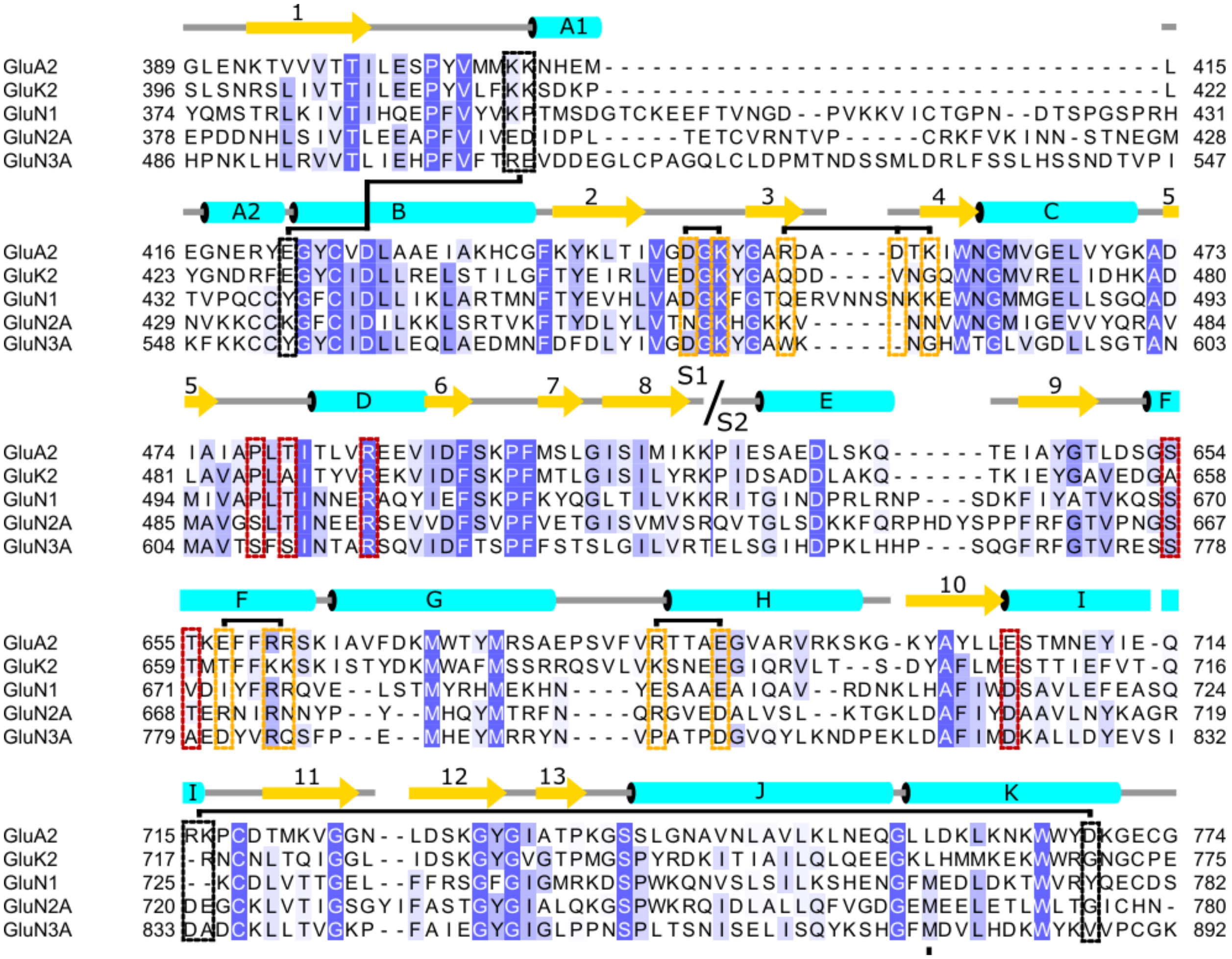

Figure S8. Sequence alignment of iGluR LBDs across AMPA, kainate, and NMDA receptors in rat. Related to Figures 1, 2, and 4. Binding pocket residues are boxed in red. Residues that make metastable interactions are boxed in yellow. Off-pathway residues that were mutated are boxed in black.

Table S1. Ligand-binding trajectories for the dimer and monomer systems. Related to Figures 1, S3, and S4, and Movies S1–S4.

	No. of ligands	Event [a]	Apo, prior [b]	ligand docked [c]	ligand conformation	(ξ1, ξ2) median[d]	Apo, post [e]	Previous Trajectory
T_{dim1}	20	A	0.93	2.49	crystallographic	11.7, 11.1	N/A	N/A
T_{dim2}	20	A	1.39	0.88	crystallographic → inverted	13.1, 11.5	N/A	N/A
T_{dim3}	20	A	N/A	3.12	inverted	12.9, 11.6	N/A	T_{dim6}
T_{dim4}	20	A	0.08	1.4	inverted	12.7, 11.2	N/A	T_{dim5}
T_{dim5}	20	D	N/A	2.49	crystallographic	11.7, 11.1	0.08	T_{dim1}
T_{dim6}	20	D	N/A	0.88	inverted	13.1, 11.5	N/A	T_{dim2}
T_{dim7}	20	D	N/A	1.4	inverted	12.7, 11.2	0.48	T_{dim4}
T_{dim8}	2	D	N/A	1.44	crystallographic	11.5, 11.1	4.98	N/A
T_{dim9}	2	D	N/A	3.57	crystallographic	11.7, 10.8	2.85	N/A
T_{mon1}	1	A	2.94	2.04	inverted	12.6, 11.1	N/A	T_{mon4}
T_{mon2}	10	A	1.25	0.78	inverted	14.1, 11.7	N/A	N/A
T_{mon3}	10	D	N/A	0.78	inverted	14.1, 11.7	0.88	T_{mon2}
T_{mon4}	1	D	N/A	1.25	crystallographic	11.1, 11.4	2.94	N/A
T_{mon5}	1	D	N/A	0.98	crystallographic	11.2, 10.7	9.32	N/A
T_{mon6}	1	D	N/A	5.52	crystallographic → inverted	12.4, 11.7	5.21	N/A
T_{mon7}	1	D	N/A	1.84	crystallographic	11.5, 11.0	7.2	N/A
T_{mon8}	1	D	N/A	1.85	crystallographic	11.8, 11.2	6.51	N/A

[a] Binding events are labeled A for association and D for dissociation
[b] μs in the apo conformation prior to the binding event
[c] μs in the docked ligand conformation
[d] units are in Ångstroms
[e] μs in the apo conformation after the binding event

Table S2. Quantities used in the calculation of k_{on} (= 1.4×10^7 M^{-1} s^{-1}). Related to STAR Methods.

No. of subunits	No. of Ligands	Free Ligand Concentration (mM)	Time spent in bulk solvent (µs)	Binding Events
1	1	3.9	31.18	1
2	1	3.5	2.13	0
2	2	6.9	2.85	0
1	9	35.1	0.78	0
1	10	38.9	2.13	1
2	18	63.5	3.43	2
2	19	67.1	1.02	1
2	20	70.6	0.93	1

Table S3. Kinetic properties of WT GluA2 and mutants. Related to Figures 4–6.

Mutant / Condition	$t_{10\text{-}90\%\ rise}$, μs (*n*)	*P*	τ_{deact}, ms (*n*)	*P*	τ_{des}, ms (*n*)	*P*	τ_{rec}, ms (*n*)	*P*
WT GluA2	190 ± 30 (4)		1.5 ± 0.2 (6)		10.4 ± 1.1 (8)		59 ± 4 (3)	
R453D	610 ± 60 (4)	0.005	3.6 ± 0.1 (3)	0.08	8.4 ± 0.6 (3)	0.2	31 ± 3 (3)	0.01
K458D	330 ± 30 (3)	0.03	1.4 ± 0.1 (3)	0.7	11.7 ± 1.3 (3)	0.5	60 ± 5 (3)	0.8
R453A, D456A, K458A (RDK-AAA)	730 ± 80 (6)	0.0003	3.7 ± 0.1 (4)	0.003	7.1 ± 0.7 (7)	0.03	32 ± 5 (3)	0.02
R660E	250 ± 10 (4)	0.2	2.4 ± 0.2 (4)	0.02	8.6 ± 0.6 (4)	0.2	75 ± 9 (3)	0.3
R661E	300 ± 30 (3)	0.07	3 ± 0.1 (3)	0.07	10.8 ± 2.3 (3)	0.9	48 ± 3 (3)	0.1
E657A, R660A, R661A (ERR-AAA)	550 ± 80 (6)	0.004	3.8 ± 0.4 (4)	0.01	7.8 ± 1.7 (6)	0.2	45 ± 9 (3)	0.3
D447A, K449A (DK-AA)	410 ± 40 (4)	0.01	3.1 ± 0.5 (3)	0.1	6.8 ± 0.8 (4)	0.03	40 ± 5 (3)	0.06
R684A, E688A (RE-AA)	590 ± 50 (5)	0.0002	4.9 ± 0.7 (3)	0.04	10.8 ± 1.3 (5)	0.8	13 ± 1 (3)	0.008
K409A, K410A, E422A (KKE-AAA)	310 ± 10 (3)	0.04	1.1 ± 0.1 (4)	0.1	11.1 ± 1.3 (4)	0.7	53 ± 8 (3)	0.6
R715A K716A D769A (RKD-AAA)	100 ± 5 (4)	0.07	2.4 ± 0.1 (4)	0.005	9 ± 0.7 (4)	0.4	28 ± 4 (3)	0.01
WT GluA2 (50 mM Glu)	180 ± 40 (3)		1.2 ± 0.1 (3)		6.6 ± 0.8 (3)			
RDK-AAA (50 mM Glu)	660 ± 70 (3)	0.01	3.7 ± 0.1 (3)	0.0003	7.7 ± 0.3 (3)	0.3		

All experiments were done with 10 mM glutamate except where noted. Activation and deactivation data are plotted in Figures 4–6. The number of experiments is indicated in brackets. $t_{10\text{-}90\%\ rise}$, rise time; τ_{deact}, time constant from single exponential fits to the deactivation decay; τ_{des}, time constant from single exponential fits to the desensitization decay; τ_{rec}, recovery time constant from Hodgkin-Huxley fits with $h = 2$ (see STAR Methods). *P* values are from two-tailed Student's *t*-test against WT values.

Table S4. Ligand-binding trajectories for the ERR-AAA mutant LBD. Related to Figure 5 and Movie S4.

	No. of ligands	Event [a]	Apo, prior [b]	ligand docked [c]	ligand conformation	(ξ1, ξ2) median[d]	Apo, post [e]	Previous Trajectory
T_{mut1}	10	A	0.06	0.31	inverted	15.3, 11.8	N/A	N/A
T_{mut2}	10	A	0.10	0.75	inverted	12.8, 11.5	N/A	T_{mut3}
T_{mut3}	10	D	N/A	0.31	inverted	15.3, 11.8	0.10	T_{mut1}
T_{mut4}	10	D	N/A	0.75	inverted	12.8, 11.5	N/A	N/A

[a] Binding events are labeled A for association and D for dissociation
[b] μs in the apo conformation prior to the binding event
[c] μs in the docked ligand conformation
[d] units are in Ångstroms
[e] μs in the apo conformation after the binding event

Movie S1. Glutamate binding in the crystallographic conformation. Related to Figure 1. The binding events shown correspond to T_{dim1}, a system consisting of the LBD dimer with 20 ligands. For clarity, the opposing subunit and ligands that do not bind are not shown.

Movie S2. Glutamate binding in the inverted conformation. Related to Figure 3 and Figure S3. The binding events shown correspond to Figure S3 and T_{mon1} in a system consisting of the LBD monomer with one ligand.

Movie S3. Glutamate converting from the crystallographic to inverted conformations within the binding pocket. Related to Figure 3 and Figure S4. The binding events shown correspond to T_{dim2}, a system consisting of the LBD dimer with 20 ligands. For clarity, the opposing subunit and ligand that do not bind are not shown.

Movie S4. Glutamate binding in the ERR-AAA mutant LBD. Related to Figure 5. The binding events shown correspond to T_{mut1}, a system consisting of the LBD monomer with 10 ligands. This movie starts with the ligand's α- and γ-carboxylates interacting with R684 and R675, respectively, on Lobe 2. Prior to this interaction, the ligand was diffusing in bulk solvent. Interactions between the ligand and Lobe 2 residues break, and the ligand diffuses into the binding pocket to contact R485. R485 initially coordinates the ligand’s α-carboxyl group, but these contacts are subsequently severed, and the ligand rotates such that R485 coordinates the γ-carboxyl group. The

ligand then transitions into the binding pocket and adopts the inverted pose. Interactions are formed between the ligand's amide nitrogen and Y450 and E705. For clarity, the ligands that do not bind are not shown.
